# Quaternary vertebrate faunas from Sumba, Indonesia: implications for Wallacean biogeography and evolution

**DOI:** 10.1098/rspb.2017.1278

**Published:** 2017-08-30

**Authors:** Samuel T. Turvey, Jennifer J. Crees, James Hansford, Timothy E. Jeffree, Nick Crumpton, Iwan Kurniawan, Erick Setiyabudi, Thomas Guillerme, Umbu Paranggarimu, Anthony Dosseto, Gerrit D. van den Bergh

**Affiliations:** 1 Institute of Zoology, Zoological Society of London, Regent's Park, London NW1 4RY, UK; 2 Ocean and Earth Science, National Oceanography Centre, University of Southampton, Southampton SO14 3ZH, UK; 3 Geology Museum Bandung, Bandung 40122, Indonesia; 4 Department of Life Sciences, Imperial College London, Silwood Park, Ascot SL5 7PY, UK; 5 KOPPESDA, Kampung Arab, Waingapu, Sumba Timur, Indonesia; 6 Wollongong Isotope Geochronology Laboratory, School of Earth and Environmental Sciences, University of Wollongong, Wollongong, New South Wales 2522, Australia; 7 Centre for Archaeological Science, School of Earth and Environmental Sciences, University of Wollongong, Wollongong, New South Wales 2522, Australia

**Keywords:** biogeography, island evolution, murid, Quaternary extinctions, *Stegodon*, Wallacea

## Abstract

Historical patterns of diversity, biogeography and faunal turnover remain poorly understood for Wallacea, the biologically and geologically complex island region between the Asian and Australian continental shelves. A distinctive Quaternary vertebrate fauna containing the small-bodied hominin *Homo floresiensis*, pygmy *Stegodon* proboscideans, varanids and giant murids has been described from Flores, but Quaternary faunas are poorly known from most other Lesser Sunda Islands. We report the discovery of extensive new fossil vertebrate collections from Pleistocene and Holocene deposits on Sumba, a large Wallacean island situated less than 50 km south of Flores. A fossil assemblage recovered from a Pleistocene deposit at Lewapaku in the interior highlands of Sumba, which may be close to 1 million years old, contains a series of skeletal elements of a very small *Stegodon* referable to *S. sumbaensis*, a tooth attributable to *Varanus komodoensis*, and fragmentary remains of unidentified giant murids. Holocene cave deposits at Mahaniwa dated to approximately 2000–3500 BP yielded extensive material of two new genera of endemic large-bodied murids, as well as fossils of an extinct frugivorous varanid. This new baseline for reconstructing Wallacean faunal histories reveals that Sumba's Quaternary vertebrate fauna, although phylogenetically distinctive, was comparable in diversity and composition to the Quaternary fauna of Flores, suggesting that similar assemblages may have characterized Quaternary terrestrial ecosystems on many or all of the larger Lesser Sunda Islands.

## 1. Introduction

Islands represent important ‘natural laboratories’ for investigating evolutionary patterns, processes and dynamics [1,2], and unusual patterns of morphological evolution are observed in island environments, including gigantism in small-bodied lineages and dwarfism in large-bodied lineages [3–5]. However, insular taxa are particularly vulnerable to human pressures, and many island systems have lost a substantial proportion of their Quaternary vertebrate faunas following prehistoric human arrival [6,7]. Reconstructing historical diversity is therefore essential to overcome this ‘extinction filter’ and understand the evolutionary origin, morphological adaptation and biogeographic affinity of island faunas.

Such research is particularly important in southeast Asia, a geologically complex region with spatially complex biodiversity and endemism, which has provided insights into biogeographic processes for over a century [1]. In particular, faunal relationships across Wallacea, the islands that were never connected with the Asian or Australian continental shelves during periods of low sea level, challenge easy interpretation [8–10]. Some vertebrate groups (e.g. proboscideans) are known to have become completely extinct in Wallacea during the Late Quaternary, possibly owing to prehistoric interactions with human colonists [4,5] or alternatively because of climatic factors or postulated long-term genetic decline [11]. Other vertebrate groups (e.g. murid rodents) still exhibit high levels of species richness and morphological disparity across the region, but the extent to which their Quaternary diversity and biogeography have been modified by past human activity remains unclear [12–14].

Interest in Quaternary vertebrate evolution in Wallacea has been raised by the discovery of a small-bodied endemic hominin, *Homo floresiensis*, from the Pleistocene of Flores in the eastern Lesser Sunda Islands [15], with hominin bones and stone artefacts dating from approximately 1.02 Ma until approximately 100–50 ka [16,17]. *H. floresiensis* coexisted with a distinctive, impoverished Quaternary insular vertebrate fauna. Late Pleistocene deposits on Flores contain the pygmy proboscidean *Stegodon florensis insularis*, the Komodo dragon *Varanus komodoensis* and a smaller extinct varanid with distinctive bunodont teeth, *Varanus hooijeri*, giant murid rodents (*Papagomys armandvillei*, *Papagomys theodorverhoeveni*, *Spelaeomys florensis* and an undescribed giant shrew rat), smaller murids (*Komodomys rintjanus*, *Paulamys naso*, *Rattus hainaldi*) and a giant stork [12,18–24]. Older Pleistocene deposits on Flores also contain a giant tortoise, another giant murid (*Hooijeromys nusatenggara*) and other *Stegodon* species [12,20,22]. All three smaller murids, one giant rat (*P. armandvillei*) and Komodo dragons still occur on Flores and/or nearby small islands [12,24–27]; *P. theodorverhoeveni*, *Sp. florensis* and *V. hooijeri* survived into the Holocene but are now extinct [20,22,23,28].

Flores has never been joined to other large islands in the Lesser Sundas, but its extant fauna shows biogeographic affinities with nearby islands [8,9], suggesting that ecologically similar Quaternary vertebrate faunas may also have had a wider former distribution across Wallacea. Distinctive extinct Quaternary vertebrates (mainly endemic *Stegodon* species, rodents and/or other small-bodied vertebrate taxa) have also been described from Sulawesi, Timor, Halmahera and some smaller Wallacean islands [4,10,13,29–34]. In particular, two *Stegodon* species, fossil varanids and a series of extinct giant murids have been reported from the Quaternary of Timor, although most of these rodent taxa have not yet been formally described [11,13,29,30,34]. However, the Quaternary record for much of Wallacea is still very poorly known, limiting our understanding of the evolutionary history and faunal relationships of the region's insular biotas [4,10]. Research into the Quaternary record of other Wallacean islands would, therefore, help to characterize patterns of past diversity and turnover in insular vertebrate faunas in relation to different island environments, as well as assisting reconstruction of regional colonization histories, evolutionary processes and island ecologies [3,22,35].

**Figure 1.**
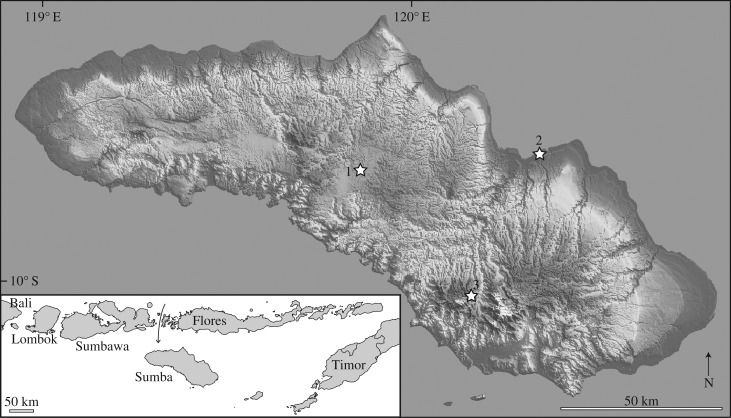
Quaternary fossil localities on Sumba. 1, Lewapaku; 2, Watumbaka; 3, Mahaniwa. Inset, location of Sumba in Lesser Sundas, showing direction of Indonesian Throughflow past Flores and nearby islands towards Sumba.

Sumba (figure 1) is a large island in the Lesser Sundas, with an area of approximately 11 000 km^2^, greater than 70% that of Timor and greater than 80% that of Flores [8]. It is a continental fragment or ‘microcontinent’ in the non-volcanic Sunda–Banda forearc system, and has never been connected to other Lesser Sunda Islands. It has occupied approximately its present position since the Miocene, and has been subaerial from the Late Pliocene [36–39]. Sumba contains several extant endemic vertebrates, including eight endemic birds [40,41], and shares other restricted-range endemics with Flores [8,40]. Mammal research conducted on Sumba has been limited [42,43], and its Quaternary history is poorly known. The only Quaternary vertebrate fossil reported from Sumba is a *Stegodon* mandible found in 1979 in a coastal terrace near Watumbaka and described as an endemic species, *S. sumbaensis* [44].

We conducted new palaeontological research on Sumba in October 2011 [45] and June–July 2014, and recovered extensive new vertebrate collections from Pleistocene and Holocene deposits. We describe these finds here in detail, and use them to refine our understanding of Quaternary evolution and biogeography in Wallacea and to provide a new baseline for reconstructing the region's faunal history.

## 2. Material and methods

### (a) Pleistocene fauna

The Watambuka type locality of *S. sumbaensis* (figure 1), where the holotype mandible was found embedded in a hard calcified sandstone and gravel deposit cropping out at the surface of a dry river bed [44], was visited in 2011. This shallow dry river valley lies at the foot slope of a 3 m high cliff face exposing a 2–3 m thick transgressive sequence of beach rock sand with reworked coral rubble capped by coral-reef limestone. No additional vertebrate fossil remains were recovered.

Vertebrate fossils were collected at Lewapaku village in 2011, from a 2.5 m deep, 2 × 2 m excavation in the backyard of a local resident (9.697° S, 119.873° E) (figure 1; electronic supplementary material, figure S1). The site is located in a 8 × 12 km wide basin at 500–520 m a.s.l. and surrounded by karst hills. The basin floor is made of horizontally bedded, well-consolidated marine sandy marl of the Late Miocene–Pliocene Waikabubak Formation [46], overlaid by a 20 cm thick sandy layer containing terrestrial vertebrate remains and then by 2.20 m of homogeneous lacustrine/paludrine clay (electronic supplementary material, figure S1). The fossil layer consists of poorly sorted coarse sandy material (carbonate fragments with local origin; pebbles up to 8 cm diameter) resting unconformably on the marine marl. Larger fossils were collected directly from the unconsolidated sediment, and the sediments of the fossil-bearing layer were wet-sieved over 2 mm mesh. A series of skeletal elements of a small *Stegodon* (apparently from multiple individuals based on ontogenetic variation between elements), a large ziphodont varanid tooth, and a rodent lower incisor, two proximal femur fragments and tibial diaphysis fragment were collected.

### (b) Holocene fauna

Vertebrate fossils were found in 2014 in two small caves in karst hills near Mahaniwa village at approximately 830 m a.s.l. (figure 1; electronic supplementary material, figure S1). Most fossils were found in Liang Lawuala cave (10.032° S, 120.168° E) in a rich surficial bone deposit in a horizontal side-passage branching back from the main downward-sloping passage approximately 10 m below the entrance. The side-passage terminates below the entrance chamber and contains a small talus cone including further fossil material that has probably fallen through crevices from the entrance. Further fossils were found in a 50 cm test-pit in the horizontal entrance chamber of nearby Liang Minabuti cave (10.029° S, 120.171° E). Vertebrate material was collected by dry sieving. Exploration of 19 other caves across Sumba (electronic supplementary material, table S1) only found unfossilized bones of native bats and invasive murids that reached the eastern Lesser Sundas in the Late Holocene [20,24].

Nearly all the Mahaniwa fossils represent an extensive sample of rodent bones. Abundant rodent mandibles and postcranial elements and a few cranial fragments (more than 3000 elements) were found in Liang Lawuala along with bat, cave swiftlet and reptile bones. We interpret this assemblage as probably deposited by both natural accumulation (bone rain) from the entrance chamber and concentration of small vertebrates by a taphonomic agent. The large quantity of rodent material and observed skeletal representation in a concentrated surficial bone layer is consistent with prey accumulation by an owl or other raptor, probably feeding in the entrance chamber [47]. Fossil material occurred at much lower density in Liang Minabuti, where accumulation is interpreted as non-predator mediated.

## 3. Results

Measurements were taken using dial callipers accurate to 0.01 mm. Rodent tooth measurements follow [23], and rodent molar crown nomenclature follows [13]. All previously undescribed fossil material reported here is accessioned in the Vertebrate Palaeontology Collection, Geology Museum Bandung.

### (a) *Stegodon sumbaensis* Sartono 1979

The holotype of *S. sumbaensis* consists of a partial left mandibular body with two molars [44]. Re-examination of the specimen by G.D.v.d.B. shows that it is an adult specimen with a total height (excluding broken condyle) of approximately 250 mm, a maximum width of the horizontal ramus of approximately 60 mm (measurement M18 [48]; estimated because medial side of ramus has superficial damage of cortical bone at the point of maximum thickness), and with the two last molars (m2–m3) present and showing dental wear stage M2–M3-A of [49] (modified in [48]; electronic supplementary material, text S1). Despite some damage to the specimen, it is clearly significantly narrower than adult mandibles of *Stegodon sondaari* from Tangi Talo on Flores with similar wear stages (e.g. MGB-TT12-TF-F80, wear stage M2–M3-A, maximum width = 74.5 mm; MGB-TT13-TG-F1, slightly younger but larger individual with wear stage M2-A (i.e. no m3 ridges showing wear), maximum width = 81 mm [5]). Dental proportions shown by *S. sondaari* are also greater than shown by the holotype of *S. sumbaensis* [5]. As *S. sondaari* was to date the smallest known well-described *Stegodon*, these measurements show that the *S. sumbaensis* type mandible clearly represents an extremely small pygmy *Stegodon* species.

**Figure 2.**
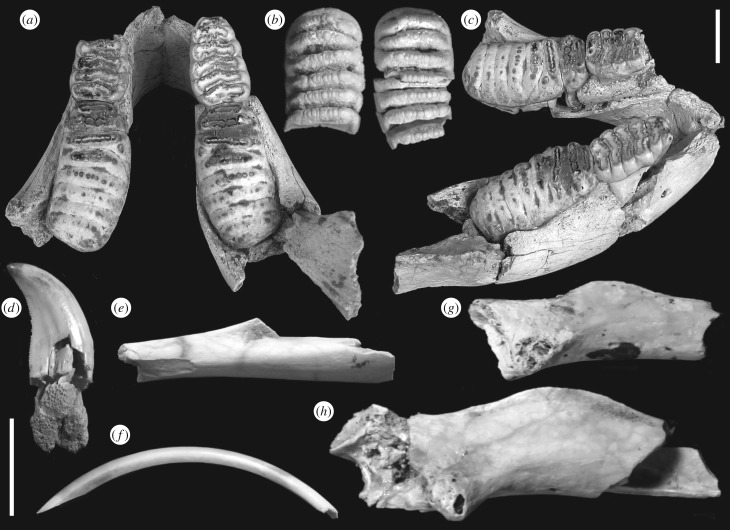
Pleistocene fossils from Lewapaku. (*a–c*) MGB-19650, *Stegodon* cf. *sumbaensis*. (*a,c*) Mandible, dorsal and lateral views; (*b*) isolated left and right m1 associated with mandible. (*d*) MGB-19665, *Varanus* cf. *komodoensis* tooth; (*e*) MGB-19670, rodent tibial diaphysis fragment; (*f*) MGB-19667, rodent lower incisor; (*g*) MGB-19669, small rodent proximal femur fragment; (*h*) MGB-19668, large rodent proximal femur fragment. (*a,b*) Scale bar, 2 cm; (*c–g*) scale bar, 1 cm.

The fossils from Lewapaku that can be attributed to a small-sized *Stegodon* are a juvenile mandible (MGB-19650), a worn molar fragment (MGB-19651), an isolated molar lamella (MGB-19652), a portion of a tusk (MGB-19653), a proximal metacarpal III fragment (MGB-19654), a distal metapodial fragment (MGB-19655), a proximal humerus fragment (MGB-19656) and various small skull fragments, three vertebra fragments and several costal fragments (figure 2; electronic supplementary material, text S1 and figure S2).

The Lewapaku juvenile mandible, representing dental wear stage dP3-dP4-A', is of very small size even given its ontogenetic stage (height of horizontal ramus measured at level of anterior border of dental alveolus = 43.8 mm; height of horizontal ramus measured at level of anterior onset of ascending ramus = 34.3 mm; total width of mandible at level of onset of ascending ramii = 108.3 mm (measurement M15 in [48]); maximum transverse diameter of dextral horizontal ramus = 40.4 mm (measurement M18); minimum transverse diameter of dextral horizontal ramus = 15.2 mm (measurement M20)). As with size comparisons for the small adult mandible from Watumbaka, the sizes of the milk molars of the juvenile mandible (right dp3 length = 29.7 mm; width = 19.9 mm; left dp4 length = 51.5 mm; width = 25.8 mm) fall just within or slightly below the size range of homologue elements of *S. sondaari* (dp3 length = 28–37.6 mm; width = 15.7–19.6 mm; dp4 length = 52.4–61.5 mm; width = 22.3–29 mm; [48] and G.D.v.d.B., unpublished data, 2017). The juvenile Lewapaku mandible corresponds in expected size based on extrapolation from growth curves to the *S. sumbaensis* holotype mandible (electronic supplementary material, figure S3), and we interpret it as probably conspecific or at least a member of the same lineage. The Lewapaku mandible is also smaller than other juvenile Wallacean *Stegodon* mandibles of similar wear stage (e.g. [20]).

The Lewapaku tusk fragment is broken at both sides and measures 180 mm long. A cone-shaped cavity of the pulpa is not present at either end, indicating that the fragment represents an intermediate tusk portion. It is slightly compressed in cross-section, with a maximum diameter of 69.5 mm and minimum diameter of 60.1 mm; compression is probably partly due to post-mortem pressure, as various longitudinal cracks are present and the broken ends exhibit a crumbly, broken mass of dentine, so that the original diameter is therefore probably close to 65 mm. The tusk fragment is comparable in size to the largest tusk fragment known of *S. sondaari* (63 mm) [50]. Four tusk fragments from the slightly larger *Stegodon timorensis* have a diameter of 81–85 mm (G.D.v.d.B., unpublished data, 2017).

### (b) Pleistocene Muridae

The Pleistocene rodent material from Lewapaku (figure 2) is very fragmentary. The lower incisor (MGB-19667) is 31 mm long from tip to broken base and 2.57 mm wide. Similar-sized lower incisors are seen in *Papagomys* on Flores [23]. One proximal femur fragment (MGB-19668) is very large, with a well-developed deltoid crest characteristic of murids. The other proximal femur fragment (MGB-19669) is smaller and more incomplete. The tibial diaphysis fragment (MGB-19670), showing the divergence of fused tibia and fibula seen in most non-sciuromorph rodents [51], is also referable to a giant murid.

### (c) *Varanus komodoensis* Ouwens 1912

The ziphodont varanid tooth from Lewapaku (MGB-19665; figure 2) can be assigned to *V. komodoensis*. Its crown is strongly compressed transversely and recurved backwards, with a sharp, delicately serrate posterior cutting edge, a height of 13.2 mm and anteroposterior diameter at crown base of 6.7 mm. Tooth size is within the range of modern *V. komodoensis* (anteroposterior diameter, 2.1–7.5 mm; crown height, 5.1–16.1 mm; *n* = 87 teeth from six individuals) [52].

### (d) Holocene Muridae

Two large-bodied murids are present in the Mahaniwa sample. These are markedly larger than introduced *R. argentiventer* and *R. rattus* from Sumba [25,43], and have distinctive morphologies that differ from other Quaternary–Recent murids previously described from southeast Asia and Australasia (electronic supplementary material, text S2). Compared with other eastern Lesser Sunda murids, neither taxon exhibits the highly cuspidate complex molars or deep robust dentary of *Spelaeomys* (Flores) or *Coryphomys* (Timor), and both have a similar higher number of molar roots (M1: 5, M2: 4, M3: 3, m1: 4, m2: 3, m3: 3) than these genera [12,13]. Instead, they show closer molar and dentary morphology to Flores murids (*Hooijeromys*, *Komodomys*, *Papagomys*, *Paulamys*), which are probably more closely related to each other than to other taxa [12].

**Figure 3.**
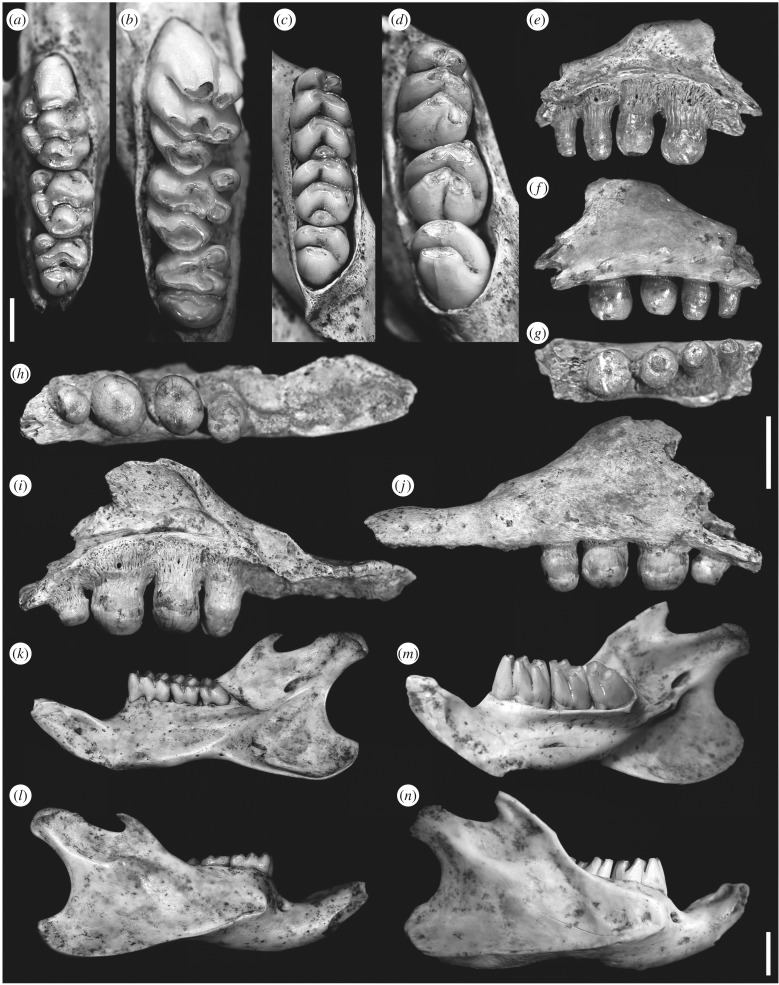
Holocene fossils from Mahaniwa. (*a,c,k–l*) *Milimonggamys juliae* gen. et sp. nov. (*a*) LL 2014/2, left maxillary toothrow; (*b*) LL 2014/1 (holotype), right mandibular toothrow; (*k–l*) LL 2014/7, right hemimandible; labial and lingual views. (*b,d,m–n*), *Raksasamys tikusbesar* gen. et sp. nov. (*b*) LL 2014/13, right maxillary toothrow; (*d*) LL 2014/17, right mandibular toothrow; (*m–n*) LL 2014/9 (holotype), right hemimandible; labial and lingual views. (*e–j*) *Varanus* cf. *hooijeri*. (*e–g*) LL 2014/21, right maxillary fragment; labial, lingual and occlusal views. (*h–j*) LL 2014/22, left maxillary fragment; occlusal, lingual and labial views. (*a–d*) Scale bar, 2 mm; (*e–j*) scale bar, 5 mm; (*k–n*) scale bar, 5 mm.

One of the new murids differs from these genera in having a posterior cingulum on M3 (absent in *Hooijeromys*, *Komodomys*, *Papagomys* and *Paulamys*); cusp t3 present on M2 and M3 (typically absent in *Papagomys* and *Paulamys*) and reduced on M1 (relatively large in *Hooijeromys* and *Komodomys*, absent in *Paulamys*); chevronate upper molar laminae (transverse in *Hooijeromys* and *Paulamys*); M2 longer than wide (unlike *Hooijeromys*); posterior margin of incisive foramina opposite anterior M1 (more anterior in *Hooijeromys*, *Papagomys* and *Paulamys*); anterolabial cuspid not disrupting transverse anterior margin of m2 (versus *Komodomys* and *Papagomys*) and posterior cingulid absent on m3 (versus *Komodomys*) [12,23–26]. Its mandible has reduced coronoid and angular processes that do not extend dorsally or posteriorly beyond the level of the articular condyle; this mandible morphology is only similar to *Komodomys* among the endemic Flores murid genera, but is otherwise similar to some southeast Asian Rattini (e.g. *Maxomys*) [53]. It is smaller than *Coryphomys*, *Hooijeromys*, *Papagomys* and *Spelaeomys* and larger than *Komodomys* and *Paulamy*s; its body mass is estimated as 251.2–336.3 g (electronic supplementary material, table S2). We describe it as the new genus and species *Milimonggamys juliae* (figure 3; electronic supplementary material, text S2 and figure S4).

The other murid is much larger than *Milimonggamys*; it is smaller than *Coryphomys* and *Spelaeomys*, larger than *Hooijeromys* and close in size to *P. theodorverhoeveni*, with a body mass estimate of 468.2–680.5 g (electronic supplementary material, table S2). This giant murid has unusual, very high-crowned and strongly fluted molars with simplified crown morphology and thin flattened upper molar laminae that distinguish it from other eastern Lesser Sunda murids. It also differs in having transverse upper laminae (chevronate in *Komodomys*, *Milimonggamys* and *Papagomys*); lacking a posterior cingulum on upper molars (versus *Milimonggamys*); having M1 with central cusps t2, t5 and t8 similar in proportion to lingual cusps t1 and t4, but labial cusps t3, t6 and t9 extremely reduced compared with *Komodomys* and *Papagomys*; cusp t3 reduced on M1 and absent on M2 and M3 (present in *Hooijeromys*, *Komodomys* and *Milimonggamys*); cusp t7 present on M3 (absent in *Hooijeromys*, *Komodomys*, *Milimonggamys*, *Papagomys* and *Paulamys*); posterior margin of incisive foramina markedly anterior to M1 (more posterior in *Komodomys* and *Milimonggamys*); postpalatal margin opposite middle of m3 (more posterior in *Hooijeromys*, *Komodomys* and *Paulamys*); m2 approximately as wide as long (versus *Milimonggamys* and *Paulamys*); anterolabial cuspid disrupts transverse anterior margin of m2 (versus *Hooijeromys*, *Milimonggamys* and *Paulamys*); lacks well-expressed anterolabial cuspid on m3 (versus *Hooijeromys*, *Komodomys*, *Milimonggamys*, *Papagomys* and *Paulamys*); and almost always lacks anterolabial cusplet, posterolabial cusplet and posterior cingulid on lower molars (versus *Komodomys*, *Milimonggamys*, *Papagomys* and *Paulamys*) [12,23–26]. It may be morphologically similar to the undescribed ‘large murid genus B’ from the Quaternary of Timor [34]; however, proper comparison must await formal description of the Timor rodent, and this taxon differs in having molar laminar tubes that enter the bony structure of the jaw, whereas in the new rodent the molar crown is still visible near the base of the lower molars in lateral profile. The mandible of the new rodent differs from that of *Milimonggamys* in having a very high ascending ramus defined by a large coronoid process, and a wider and longer angular process; this morphology is much more similar to that seen in endemic Flores murids as well as ‘large murid genus B’ from Timor and other large-bodied southeast Asian murids (e.g. *Lenomys*, *Mallomys*) [12,34]. We describe this distinctive giant murid as the new genus and species *Raksasamys tikusbesar* (figure 3; electronic supplementary material, text S2 and figure S5).

Phylogenetic analysis was conducted to investigate the affinities of *Milimonggamys* and *Raksasamys* within the biogeographically complex wider murid radiations of southeast Asia and Australasia, using a comparative taxonomic sample of six other endemic murids from the Lesser Sunda Islands (*Hooijeromys*, *Komodomys*, *Papagomys*, *Paulamys*, *R. hainaldi*, *R. timorensis*) and 17 other genera of Rattini, Hydromyini and Phloeomyini from the Sunda Shelf, the Philippines, Wallacea and Sahul (*Anisomys*, *Bunomys*, *Halmaheramys*, *Hydromys*, *Hyomys*, *Lenothrix*, *Leptomys*, *Macruromys*, *Mallomys*, *Maxomys*, *Parahydromys*, *Paruromys*, *Phloeomys*, *Pogonomys*, *Sundamys*, *Taeromys*, *Uromys*), and employing a matrix of 5966 characters (25 cranial, mandibular and dental morphological characters, and molecular data for four gene matrices taken from Fabre *et al.* [14]) (electronic supplementary material, text S3 and table S3). The phylogenetic placement of both *Milimonggamys* and *Raksasamys* in the majority consensus trees generated in these analyses was poorly resolved; only morphological data are available for these taxa, and poor resolution is expected in investigations of murid relationships using morphological data due to homoplasy [13]. However, the majority consensus molecular + morphology tree placed *Milimonggamys*, *Raksasamys*, and all six other Lesser Sunda murids within the Rattini, and the separate parsimony and Bayesian analyses of the morphology-only dataset both returned a sister-group relationship for *Raksasamys* + *Papagomys* (electronic supplementary material, figure S6).

### (e) *Varanus hooijeri* Brongersma 1958

Two varanid maxillaries (LL 2014/21–LL 2014/22; figure 3), each with four teeth, were also recovered at Liang Lawuala. Teeth on both elements show the cylindrical shape and blunt rounded bunodont crowns characteristic of posterior teeth of the extinct *V. hooijeri* from Flores [18] and are otherwise only comparable to the extant frugivorous *Varanus bitatawa* and *Varanus olivaceus* from Luzon, Philippines [54,55]*.* Tooth measurements are slightly smaller than for two *V. hooijeri* specimens from Flores; however, teeth in both Sumba maxillaries and both described Flores maxillaries also vary in size, probably reflecting ontogenetic or intraspecific variation [18] (electronic supplementary material, table S4). We assign both elements to *Varanus* cf. *hooijeri*, revealing a probable range extension for this extinct species, formerly considered endemic to Flores.

### (f) Age estimates of the fossils

The holotype of *S. sumbaensis* originates from a fluvial layer at Watumbaka that postdates the deposition, emergence and erosion of the lowest marine Terrace Complex I at Cape Laundi (because the fluvial deposits from which the *Stegodon* mandible was recovered have cut down into Terrace Complex I, they must be younger in age than the latter). Based on elevation of approximately 20 m a.s.l., this sequence can be correlated with the lowest of the dated coral-reef terrace complexes near Cape Laundi (Terrace Complex I, at 3–20 m a.s.l.); U–Th dates indicate that this terrace complex was formed during OI Stage 5, with coral ages corresponding to the three main high sea-levels at 86 kyr BP, 105 kyr BP and 125 kyr BP [36,37]. As the terrace complex is polycyclic in nature, the lack of information about the exact stratigraphic origin of the *S. sumbaensis* holotype within the Watumbaka outcrop precludes precise age estimation for this fossil, other than that it probably has a Late Pleistocene age younger than 125 kyr.

Fossils at Lewapaku originate from a clayey layer that rests unconformably on top of the deep marine sequence of the Waikabubak Formation. This marine sequence is also unconformably overlaid by a series of Quaternary coral-reef terraces along the northern edge of the island at Cape Laundi, which reach elevations of up to 475 m a.s.l.; these terraces have been extensively studied and dated by U–Th dating, and document the youngest uplift phase of Sumba [36,37]. Uranium-series dating on a *Stegodon* molar fragment from the Lewapaku assemblage (MGB-19652) did not produce reliable results; the sample has experienced extensive uranium loss, possibly because the fossil layer at Lewapaku is an aquifer (electronic supplementary material, text S1, figures S7, S8 and tables S5, S6). However, approximate age estimation of the Lewapaku site is possible by comparison of the elevation of Quaternary terraces. At an elevation of 500–520 m a.s.l., the Lewapaku Basin is approximately 40 m higher than the highest coral-reef terrace, which has been correlated with Oxygen Isotope Stage 27 (approx. 0.99 Ma) [56]. The fossil-bearing layer that rests on the Waikabubak Formation must therefore be younger than approximately 1 Ma. However, it is difficult to establish whether the fossil-bearing terrestrial layer began to accumulate directly following emergence of the basin, or at a later stage.

The Liang Lawuala and Liang Minabuti deposits contain characteristic Neolithic markers (pottery fragments, pig bones), indicating a likely mid–late Holocene age. A small amount (less than 1%) of murid postcrania at Liang Lawuala show signs of burning, suggesting possible consumption by humans (although see [33] for alternative potential explanations for burning on rodent bones). Bone samples from Liang Lawuala were submitted for accelerator mass spectrometer (AMS) ^14^C dating at the ^14^CHRONO Centre, Queen's University, Belfast (UK), and calibrated with OxCal 4.2 [57]. Three AMS ^14^C dates were obtained for Liang Lawuala: a date of 2167 ± 24 BP (=279–119 calBC) from charcoal from 5–10 cm depth (UBA-30134), a direct date of 1889 ± 33 BP (=54–222 calAD) from a *Milimonggamys* mandible from 0–5 cm depth (UBA-30135) and a direct date of 3507 ± 38 BP (=1935–1700 calBC) from a large rodent scapula surface find probably referable to *Raksasamys* (UBA-30138). Two further rodent specimens (*Raksasamys* femur, mandible; UBA-30135, 30136) did not return AMS dates because of insufficient collagen yield.

## 4. Discussion

Our fossil finds provide the first detailed understanding of Quaternary vertebrate diversity and turnover on Sumba, a large Indonesian island for which the terrestrial fossil record was almost totally unknown, and which is situated in a biogeographically complex and important region. We reveal that Sumba's Quaternary vertebrate fauna was similar in diversity and composition to the unusual ‘unbalanced’ fauna on Flores, containing not only a pygmy *Stegodon* but also Komodo dragons, another unusual and apparently regionally endemic varanid, and large-bodied murids. It is therefore likely that similar assemblages probably characterized Quaternary terrestrial ecosystems on many or all of the larger Lesser Sunda Islands. The poor available Quaternary record for most southeast Asian islands continues to hinder rigorous comparison of inter-island faunal evolution and dispersal across this biogeographically complex region, and it is possible that taxa currently represented in modern or Quaternary faunas from single Wallacean islands may have had much wider former distributions which have not yet been adequately sampled (cf. [58]). Future work on Quaternary microvertebrate faunas from Sumba may also provide important novel insights into regional biogeography. However, our new baseline of past diversity on Sumba allows new assessment of the island's biogeographic affinities.

*V. komodoensis* now has a relict distribution on Flores and nearby islands (Komodo, Rinca, Gili Motang, Padar), but had a wide Pliocene–Pleistocene distribution across both sides of Wallace's Line, from Java to Australia [52]. This cosmopolitan distribution limits its ability to provide a biogeographic signal. The species is currently unknown from the Quaternary record of Timor, where a distinctive unnamed larger extinct varanid has instead been recorded [52]; however, this apparent absence may merely reflect limited sampling in this understudied region. Conversely, *V. hooijeri* is otherwise known only from the Quaternary record of Flores [18,20,22], giving a potential signal of biogeographic affinity between Flores and Sumba.

*Milimonggamys* and *Raksasamys* both share some dental and mandibular characteristics with endemic murid genera from Flores and Timor, but exhibit other similarities to some other southeast Asian murids (electronic supplementary material, text S2 and table S3). Our phylogenetic analyses of the affinities of *Milimonggamys* and *Raksasamys* generated trees that were poorly resolved beyond being able to place these taxa and the other sampled Lesser Sunda murids within the Rattini. This poor resolution is probably due to the limited morphological character data that were available for both new taxa, and to recognized problems of widespread homoplasy in murid craniodental characters [13]. Fuller understanding of regional Quaternary biogeography and evolution must also await description and phylogenetic analysis of Timor's extinct rodent fauna [13,34], and future attempts to extract ancient DNA from Quaternary rodent material from the Lesser Sundas, which may not be feasible because of their preservation under tropical environmental conditions. Rigorous assessment of phylogenetic and biogeographic affinities of Sumba's Quaternary rodent fauna, and whether its endemic rodents represent a single within-island evolutionary radiation or multiple overwater colonizations from one or more islands, is thus not yet possible. However, both of our morphology-only phylogenetic analyses returned a sister-group relationship for *Raksasamys* + *Papagomys*, providing some support for a close biogeographic relationship between the endemic murids of Sumba and Flores, although in the absence of comparative character data for any endemic Timor rodents other than *R. timorensis*.

Sumba is situated less than 50 km south of Flores, and extensive inter-ocean transport from the Pacific Ocean to the Indian Ocean via the Indonesian Throughflow, a major crossroads of global ocean circulation, occurs southwards past Flores through the Sape and Sumba straits immediately north of Sumba [59]. Overwater ‘waif dispersal’ from Flores to Sumba is therefore a biologically plausible chance event, providing a likely explanation for the apparent biogeographic affinity seen between some components of the described Quaternary faunas from these islands. However, observed differences in size and diversity in different taxa between Sumba, Flores and other Lesser Sunda Islands—notably lower rodent species richness and maximum body size on Sumba versus Flores and Timor, smaller size of *Stegodon sumbaensis* compared with most other *Stegodon* species, and possibly also inter-island differences in varanid faunas—may be explicable on biogeographic grounds and parallel evolution rather than close regional evolutionary history, as these patterns are consistent with body size trends and species richness increasing with land area in other island systems [3,5].

Discovery of Pleistocene and Holocene faunas on Sumba that may span much or most of the last 1 Myr, including taxa directly dated to the Late Holocene, provides a temporal framework to reconstruct Quaternary vertebrate turnover compared with faunal sequences for Flores. If the Lewapaku deposit is close to 1 million years old based on correlation with coral-reef terraces at Cape Laundi, this provides a similar first occurrence for pygmy *Stegodon* and *V. komodoensis* on Sumba to the oldest record of these taxa on Flores; rodents first appear on Flores at approximately 800 ka, although this is probably a taphonomic artefact rather than true absence from older horizons [16,22,60,61]. However, the lack of dated Pleistocene deposits on Sumba prevents assessment of whether subsequent faunal turnover occurred at the Early to Middle Pleistocene transition as seen on Flores [22,60,61]. *St. sumbaensis* and *V. komodoensis* would not be expected to occur in an owl roost deposit, so that their absence from the Mahaniwa fossil sample cannot help to constrain the likely extinction timing for either species. The limited, non-diagnostic rodent material from Lewapaku also prevents assessment of whether Sumba's Pleistocene murids were part of the same endemic lineage as *Milimonggamys* and *Raksasamys*; phylogenetic continuity is likely, but local extinction and recolonization by different rodent lineages during the Late Quaternary is also possible, a phenomenon also shown regionally by stratigraphic turnover of different colonizing *Stegodon* lineages on Flores [20,22].

Sumba and Flores also both retained large murids and *Varanus* cf. *hooijeri* into the late Holocene [20,22,23]. The current status of Sumba's endemic murids is unknown, as little mammal survey work has been conducted on the island. *P. theodorverhoeveni* and *Sp. florensis* died out in the Holocene on Flores, and *Milimonggamys* and *Raksasamys* may now also be extinct because of habitat loss, hunting and/or competition with introduced species in recent millennia. However, one giant murid, *P. armandvillei*, survives on Flores. Most of Sumba's forests have been burnt or cleared for agriculture or firewood extraction and replaced by grassland [62], but Sumba has a much lower human population density than Flores and has experienced almost no human population growth since the mid-nineteenth century [8], suggesting that anthropogenic pressures may not have been so severe as to drive extinction of the native murids. Indeed, local people in Mahaniwa and elsewhere on Sumba reported that ‘giant rats’ larger than *R. rattus* still lived in the island's forests, and large rodents feature in local myths recounted to us during fieldwork in 2014. Southeast Asia contains the world's highest levels of globally threatened mammals [63], and further fieldwork to assess the possible survival of *Milimonggamys* or *Raksasamys* could represent an important conservation priority.

Our research constitutes an important new step towards clarifying the evolutionary history and Quaternary diversity of the Wallacean vertebrate fauna, and future palaeontological research on Sumba will no doubt provide further insights into vertebrate evolution in the Lesser Sundas. Whereas most other components of Flores' Quaternary vertebrate fauna were found at Liang Bua cave in the mid-twentieth century, excavations took place at this site for almost 50 years before discovery of *H. floresiensis* [4]. We therefore consider it likely that new vertebrate taxa remain to be discovered in Sumba's Quaternary fossil record. Most intriguingly, given other similarities between the distinctive Quaternary faunas from Sumba and Flores, there is no obvious biogeographic reason to suppose that regionally endemic hominin species would have been present on Flores but not Sumba. Indeed, the possibility of ancient hominin occurrence on Sumba has already been raised by the apparent presence of Palaeolithic artefacts in Pleistocene sediments [64]. We strongly encourage further investigation of Sumba's Quaternary preservational environments, which may well provide exciting new lessons for understanding both island biogeography and human evolution.

## Supplementary Material

Text S1; Text S2; Text S3; Table S1; Table S2; Table S3; Table S4; Table S5 and Table S6

## Supplementary Material

Figure S1

## Supplementary Material

Figure S2

## Supplementary Material

Figure S3

## Supplementary Material

Figure S4

## Supplementary Material

Figure S5

## Supplementary Material

Figure S6

## Supplementary Material

Figure S7

## Supplementary Material

Figure S8

## Data accessibility

The data supporting this article are described in full in the electronic supplementary material.
