# Supplementary material for: Quaternary vertebrate faunas from Sumba, Indonesia: implications for Wallacean biogeography and evolution
Source: Proc Biol Sci. 2017 Aug 30;284(1861):20171278. doi: 10.1098/rspb.2017.1278 (PMC5577490; doi:10.1098/rspb.2017.1278)
Supplement: Text S1; Text S2; Text S3; Table S1; Table S2; Table S3; Table S4; Table S5 and Table S6 [file rspb20171278supp1.docx]

**SUPPLEMENTARY INFORMATION**

**Text S1: Fossils and dating of *Stegodon sumbaensis***

**(a) Holotype**

As noted by Hooijer (1981), the measurements given by Sartono (1979) for the holotype of *Stegodon sumbaensis* do not make sense. For example, the width of the molar ridges was said by Sartono (1979) to vary between 25-27 mm; it is therefore likely that anteroposterior basal distances of the ridges were reported as transverse widths. The fossil, which is housed at the National Centre for Archaeological Research in Jakarta, was re-examined by GDvdB. The fossil comprises a left mandibular ramus of which the coronoid process, the condyle and the symphysis are broken. The broken edges are rounded and the fossil appears to be water-worn. There remains a posterior fragment of a very worn molar at the front of the ramus, followed by a molar with at least x8- ridges exposed, of which the first three are slightly worn. The posterior portion of the molar is concealed inside the alveole and the molar has an unknown number of hidden ridges. On the anterior worn molar, only the two penultimate ridges and a posterior half-ridge are preserved sufficiently to allow estimation of their basal transverse width, which varies between 36 and 33 mm for the penultimate ridge and the posterior half-ridge respectively. On the succeeding molar, the basal transverse width of the anterior four ridges increases from 33.7 mm in ridge 1 to 39.4 mm in ridge 4, and the basal width of ridge 5 can be estimated at ~40 mm. This value must be close to the maximum width, which usually occurs at the fourth or fifth ridge in the last two molars of *Stegodon* species (Hooijer 1957; van den Bergh 1999). The posterior molar has a minimum length of 76+ mm from the anterior border to the point where it disappears in the alveole. The lamellar frequency (LF; number of ridges along 10 cm longitudinal distance) is 10.0. The limited increase in basal width from the anterior molar (slightly over 36 mm) to the posterior molar (~40 mm) indicates that these represent the two last molars (m2 and m3), as was originally considered by Sartono (1979).

**(b) Additional description of *Stegodon* fossils from Lewapaku**

**Mandible** (MGB-19650) Juvenile, with both rami, worn dp3s and slightly worn dp4s in place, and m1s still under formation inside the alveoli. The anterior part of the left dp3 is broken, and it was possibly already shed during life. The right dp3 is complete, and the horizontal ramus of the right side is better preserved. The symphysis is present. The total length of the dextral dP3 is 29.7 mm. The wear figures of the ridges are folded in V-shaped patterns and point in an anterior direction in occlusal view. The entire wear surface has a pear-shaped outline, and there are x5 ridges, all worn. The maximum width of 19.9 mm is in the posteriormost ridge. The dp3 does not exhibit the typical anterior constriction usually developed in *Stegodon* lower dp3s. The LF is 17.6. The enamel is double layered and 1.0-1.4 mm thick, with the inner enamel layer brownish and the outer enamel layer white in colour.

The left dP4 of the same mandible is 51.5 mm long and bears x7x ridges, of which the anterior four are worn. The maximum width of 25.8 mm occurs at ridge 5. The LF is 16.4 and the enamel thickness (ET) varies between 1.3 and 1.6 mm thick. There is a well-developed basal pillar rising up from the valley base between ridges 2 and 3 lingually. It is 12.8 mm high and not yet modified by wear; the first ridge has a prominent medial cleft so that the wear figure is not yet completely connected. The unworn height of the posterior ridges varies between 18.5 and 19.6 mm. The H/W (height/width) indices of ridges 4-6 vary between 72.8 and 76.7.

The posterior portion of both m1s is missing. Seven ridges of the right m1 are preserved. The m1 was still under formation when the animal died, and there may have been more than seven ridges present in a completed m1. The enamel of ridges 1-3 is fully formed, and these ridges are fully fused at their bases. The lower portion of the posterior part of ridge 4 was not yet mineralized when the animal died. Of the subsequently more posterior ridges the enamel was even less developed, and only one-third of ridge 7 was mineralized. Deposition of cement had started between ridges 1 and 2. Ridge 4 has the greatest width of 27.6 mm, but as this ridge is not yet fully formed, the width in a fully formed m1 might have been slightly larger. The height of ridges 2 and 3 are 17.6 and 15.9 mm respectively, and the H/W indices are 66.4 and 62.4 respectively. The LF of the anterior three ridges is 13.8.

Some measurements taken on the mandible are as follows. Height of the horizontal ramus measured at the level of the anterior border of the dental alveolus is 43.8 mm. The minimum height of the horizontal ramus measured at the level of the anterior onset of the ascending ramus (measurement M6 of van den Bergh 1999) is 34.3 mm. The total width of the mandible at the level of the onset of the ascending ramii is 108.3 mm (measurement M15 of van den Bergh 1999). The maximum transverse diameter of a single horizontal ramus (dextral) is 40.4 mm (measurement M18 of van den Bergh 1999). The minimum transverse diameter of the dextral horizontal ramus is 15.2 mm (measurement M20 of van den Bergh 1999).

From these measurements it is apparent that the mandible, even though it is from a juvenile individual, is of very small size. The size of the anterior molars falls within or below the size range of homologue elements of *Stegodon sondaari* from the Early Pleistocene site of Tangi Talo on Flores. *S. sondaari* is to date the smallest *Stegodon* known (L dp3 = 28-37.6 mm (n=2), W dp3 = 15.7-19.6 mm (n=3), L dp4 = 52.4-61.5 mm (n=3); W dp4 = 22.3-29.0 mm (n=6)). The dental wear age stage of the Lewapaku mandible is dP3-dP4-A'. Compared to two juvenile *Stegodon florensis insularis* mandibles from Liang Bua in a similar wear stage, the Lewapaku mandible appears to be smaller (figure S3).

- **Molar fragments** An isolated molar ridge (MGB-19652), unworn, measures 33.0 mm in transverse direction and has a height of 27.6 mm. Its anteroposterior thickness at the base is 9.05 mm, which translates to a LF of 11. Its larger transverse width as compared to the m1 from the mandible described above, and the lower LF, indicates that the ridge belonged to one of the last two molars, upper or lower.
- A second, completely worn molar fragment (MGB-19651) has enamel preserved on only one side, either the buccal or the lingual side. The only measurement that could be taken is the enamel thickness, which measures 2.8 mm.
- **Metapodials** The proximal fragment of a dextral metacarp III (MGB-19654) lacks the distal epiphysis, which was not yet fused and thus indicates a juvenile individual (figure S2). The fossil is superficially damaged by water wear, not preserving detailed boundaries of the various articulation facets. The proximal transverse diameter is 37.1 mm, and the anteroposterior diameter is 46 mm. The minimum transverse diameter of the diaphysis measures 30 mm, and its minimum anteroposterior diameter measures 24.9 mm.
- A distal metapodial fragment (MGB-19655) including the diaphysis is rather damaged superficially. The epiphysis is fully fused in this specimen. The minimum anteroposterior diameter of the diaphysis measures 18.2 mm, and the minimum transverse diameter measures 27.8 mm.

**Humerus** (MGB-19656) A proximal humerus epiphysis fragment belonged to an individual that was not fully grown and which had an unfused epiphysis (figure S2). The anteroposterior diameter of the caput measures ~89 mm, and the maximum transverse diameter measures 124 mm. Unfortunately, no humeri of *S. sondaari* or *S. timorensis* are available for comparison.

The remaining *Stegodon* specimens from Lewapaku, such as costa and vertebra fragments, are too damaged to take useful measurements.

**(c) Attempt at uranium-series dating**

Uranium-series dating of a *Stegodon* molar fragment from Lewapaku was undertaken by laser ablation multi-collector ICP-MS at the Wollongong Isotope Geochronology Laboratory, University of Wollongong. Laser ablation was performed with a New Wave Research 193 nm ArF excimer laser, equipped with a TV2 cell. The cut tooth fragment was ablated with a laser pulse rate of 20 Hz and a fluence of 2.7 J/cm^2^. Helium was used as a carrier gas at a flow rate of 0.9 L/min. Thorium (^230^Th, ^232^Th) and uranium (^234^U, ^235^U, ^238^U) isotopes were measured on a Thermo Neptune Plus multi-collector ICP-MS. All five isotopes were collected in static mode, with ^230^Th and ^234^U collected in ion counters. Helium flow rate and plasma parameters were tuned with NIST610 element standard to derive a ^232^Th/^238^U ratio for this standard greater than 0.8 and minimise differences in fractionation between Th and U (Bernal et al. 2005).

Measured ^234^U/^238^U, ^230^Th/^238^U and ^232^Th/^238^U isotopic ratios were corrected for elemental fractionation and Faraday cup/SEM yield by comparing measured ratios to those of a 206ka-old coral characterised independently by solution analysis. Analysis of a phosphate reference material for which isotope ratios were determined independently shows that using a coral as primary standard yields results within error of solution analyses (unpub. data). Uranium and Th concentrations were determined using NIST612 glass as calibration standard. Background subtraction and calculations of corrected ratios and concentration were performed using Iolite™. Accuracy was assessed using a 124ka-old coral also characterised independently by solution analysis.

Analysis of 18 lines ~620 μm long was carried out with a 150 μm spot size and a scan speed of 5 μm/sec (figure S7, table S5). For comparison, 18 spot analyses were also performed on a parallel transect (same pulse rate, fluence and spot size; ablation duration = 60 sec; figure S7, table S6).

Both line and spot analyses of the 124ka-old coral (MK16) yielded (^234^U/^238^U) and (^230^Th/^238^U) ratios within error of values determined by solution analysis (1.110 ± 0.002 and 0.764 ± 0.007, respectively). Spot and line analyses of the sample showed similar U concentrations and (^234^U/^238^U) activity ratios (figure S8). Differences can be attributed heterogeneity in the tooth, as (i) lines and spots were not ablated at exactly the same location, and (ii) lines represent larger areas than spots, thus averaging heterogeneity. Spot analyses yielded (^230^Th/^238^U) activity ratios systematically greater than those from line analyses, for values >1. This difference, where there is good agreement in (^234^U/^238^U), could be explained by downhole fractionation between Th and U during spot analyses (which does not affect U isotopes).

The iDAD model of Sambridge et al. (2012) and Grün et al. (2014) was used to attempt to derive an age of U uptake, and by inference, a minimum age for the tooth. The (^230^Th/^234^U) activity ratios greater than 1 in dentine and light-coloured enamel (not shown) indicated that these regions of the tooth have experienced significant U loss, to such extent that U-series data for these regions cannot be used to calculate an age. Uranium-series isotope ratios in dark enamel were used to calculate a model age. Using line analyses (n=4), we obtained an age of 165 +57/-54 ka (D/R = 1.55x10^-9^ cm^2^/s). Using spot analyses (n=5), we obtained an age of 125 ± 31 ka (D/R = 7.49x10^-13^ cm^2^/s). However, these values need to be considered with caution (at best), considering the small number of analyses used and the complex history of U mobility in this sample.

**Text S2: Systematic palaeontology**

Rodentia Bowditch 1821

Muroidea Illiger 1811

Muridae Illiger 1811

Murinae Illiger 1811

Rattini Burnett 1830

**Genus *Milimonggamys* gen. nov.**

**Type species:** *Milimonggamys juliae* gen. et sp. nov.

**Etymology:** After the milimongga, an apparently legendary animal from Sumbanese folklore (Forth 2008) that was regularly talked about by local people in East Sumba Regency during our visit in 2014, combined with “*mys*”, the standard suffix for mouse.

**Diagnosis:** Large murine with high-crowned dentition, with anteriorly curved chevronate or transverse laminae separated by deep clefts and made up of adpressed, mainly teardrop-shaped cusps; upper and lower molars all longer than wide; all upper molars lacking cusp t7; M2 and M3 lacking cusp t2; cusp t3 reduced on M1, and present on M2 and M3; M3 with posterior cingulum; M1 with five roots, M2 with four roots, M3 with three roots; m1 with narrow anteroconid with anterolabial and anterolingual cuspids separated by distinct anterior groove, and lacking anterocentral cuspid; small anterolabial cusplet sometimes present on m1; posterolabial cusplets and posterior cingulids present on m1 and m2 but not m3; transverse anterior margin of m2 not disrupted by anteriorly extending anterolabial cuspid; m1 with four roots, m2 and m3 each with three roots; posterior margin of incisive foramina and posterior margin of anterior root of zygomatic arch both situated opposite anterior M1; coronoid and angular processes reduced.

*Milimonggamys* differs from other southeast Asian and Australasian murids distributed outside the Lesser Sundas according to the following characters:

Differs from *Anisomys* (New Guinea) in having elongate incisive foramina that penetrate far into the maxilla, in having the posterior margin of the zygomatic arch opposite the occlusal surface of M1, in having a reduced rather than an enlarged posterior cingulum on upper molars, in lacking cusp t7 on upper molars, in having M2 with equal length and width, in having anterolabial cuspids and accessory labial cusplets on lower molars, in having a coronoid process that does not extend dorsally beyond the articular condyle, in having a deep posterior margin between the articular condyle and the angular process, in having an angular process tilted to form a moderately wide internal shelf, and in having the mandibular foramen dorsal to the alveolar shelf.

Differs from *Bunomys* (Sulawesi) in having only slightly slanted and overlapping cusps on upper molars, in having cusp t9 nearly incorporated into much larger cusp t8 on M1 and M2, in having cusp t3 present on M2 and M3, in having a coronoid process that does not extend dorsally beyond the articular condyle, in having an angular process tilted to form a moderately wide internal shelf, and in having the mandibular foramen dorsal to the alveolar shelf.

Differs from *Halmaheramys* (Halmahera) in having the posterior margin of the zygomatic arch opposite the occlusal surface of M1, in having only slightly slanted and overlapping cusps on upper molars, in having M2 with equal length and width, in having cusp t3 present on M2 and M3, in having large and discrete anterolabial and anterolingual cusps on m1, in having an anterolabial cuspid on m2 that does not disrupt the anterior margin, and in having a coronoid process that does not extend dorsally beyond the articular condyle.

Differs from *Hydromys* (New Guinea) in having elongate incisive foramina that penetrate far into the maxilla, in having three rather than two upper and lower molars present, in lacking cusp t7 on upper molars, in having M2 with equal length and width, in having anterolabial cuspids on lower molars, in having a coronoid process that does not extend dorsally beyond the articular condyle, and in having the mandibular foramen dorsal to the alveolar shelf.

Differs from *Hyomys* (New Guinea) in having elongate incisive foramina that penetrate far into the maxilla, in having brachydont rather than hypsodont dentition, in having small upper and lower third molars, in having only slightly slanted and overlapping cusps on upper molars, in having a reduced rather than an enlarged posterior cingulum on upper molars, in lacking cusp t7 on upper molars, in having cusp t9 nearly incorporated into much larger cusp t8 on M1 and M2, in having an anterolabial cuspid on m2 that does not disrupt the anterior margin, in having a coronoid process that does not extend dorsally beyond the articular condyle, in having a deep posterior margin between the articular condyle and the angular process, in having the mandibular foramen dorsal to the alveolar shelf, and in having a distinct incisor alveolus capsule defined by a high bulge.

Differs from *Lenothrix* (peninsular Malaysia, Borneo) in having the posterior margin of the zygomatic arch opposite the occlusal surface of M1, in having cusps strongly joined on upper and lower molars, in having a reduced rather than an enlarged posterior cingulum on upper molars, in lacking cusp t7 on upper molars, in having cusp t9 nearly incorporated into much larger cusp t8 on M1 and M2, in lacking a small accessory labial cusp behind cusp t6 on M1 and M2, in having M2 with equal length and width, in having cusp t3 present on M2 and M3, in having an anterolabial cuspid on m2 that does not disrupt the anterior margin, in having a coronoid process that does not extend dorsally beyond the articular condyle, and in having a distinct incisor alveolus capsule defined by a high bulge.

Differs from *Leptomys* (New Guinea) in lacking cusp t7 on upper molars, in having cusp t4 strongly connected to cusp t5 in M1, in having M2 with equal length and width, in having cusp t3 present on M2 and M3, in having anterolabial cuspids on lower molars, in having an angular process tilted to form a moderately wide internal shelf, and in having a distinct incisor alveolus capsule defined by a high bulge.

Differs from *Macruromys* (New Guinea) in having elongate incisive foramina that penetrate far into the maxilla, in having the posterior margin of the zygomatic arch opposite the occlusal surface of M1, in lacking cusp t7 on upper molars, in having cusp t4 strongly connected to cusp t5 in M1, in having cusp t9 nearly incorporated into much larger cusp t8 on M1 and M2, in having M2 with equal length and width, in having cusp t3 present on M2 and M3, in having anterolabial cuspids on lower molars, in having a coronoid process that does not extend dorsally beyond the articular condyle, in having a deep posterior margin between the articular condyle and the angular process, in having the mandibular foramen dorsal to the alveolar shelf, and in having a distinct incisor alveolus capsule defined by a high bulge.

Differs from *Mallomys* (New Guinea) in having brachydont rather than hypsodont dentition, in having small upper and lower third molars, in having cusps strongly joined on upper and lower molars, in having only slightly slanted and overlapping cusps on upper molars, in lacking cusp t7 on upper molars, in having cusp t4 strongly connected to cusp t5 in M1, in lacking a small accessory labial cusp behind cusp t6 on M1 and M2, in having cusp t3 present on M2 and M3, in having anterolabial cuspids and accessory labial cusplets on lower molars, in having large and discrete anterolabial and anterolingual cusps on m1, in having a coronoid process that does not extend dorsally beyond the articular condyle, in having a deep posterior margin between the articular condyle and the angular process, in having the mandibular foramen dorsal to the alveolar shelf, and in having a distinct incisor alveolus capsule defined by a high bulge.

Differs from *Maxomys* (peninsular southeast Asia, Borneo, Sumatra, Java, Palawan, Sulawesi) in having elongate incisive foramina that penetrate far into the maxilla, in having only slightly slanted and overlapping cusps on upper molars, in having M2 with equal length and width, in having cusp t3 present on M2 and M3, in having anterolabial cuspids and accessory labial cusplets on lower molars, in having large and discrete anterolabial and anterolingual cusps on m1, in having a coronoid process that does not extend dorsally beyond the articular condyle, in having a deep posterior margin between the articular condyle and the angular process, and in having a distinct incisor alveolus capsule defined by a high bulge.

Differs from *Parahydromys* (New Guinea) in having elongate incisive foramina that penetrate far into the maxilla, in lacking cusp t7 on upper molars, in having anterolabial cuspids and accessory labial cusplets on lower molars, in having a coronoid process that does not extend dorsally beyond the articular condyle, in having an angular process tilted to form a moderately wide internal shelf, in having a distinct incisor alveolus capsule defined by a high bulge, and in having three rather than two upper and lower molars present.

Differs from *Paruromys* (Sulawesi) in having elongate incisive foramina that penetrate far into the maxilla, in having brachydont rather than hypsodont dentition, in lacking cusp t7 on upper molars, in having cusp t4 strongly connected to cusp t5 in M1, in having cusp t9 nearly incorporated into much larger cusp t8 on M1 and M2, in having a coronoid process that does not extend dorsally beyond the articular condyle, and in having an angular process tilted to form a moderately wide internal shelf.

Differs from *Phloeomys* (Philippines) in having elongate incisive foramina that penetrate far into the maxilla, in having brachydont rather than hypsodont dentition, in lacking cusp t7 on upper molars, in having M2 with equal length and width, in having cusp t3 present on M2 and M3, in having anterolabial cuspids and accessory labial cusplets on lower molars, in having a deep posterior margin between the articular condyle and the angular process, in having the mandibular foramen dorsal to the alveolar shelf, and in having a distinct incisor alveolus capsule defined by a high bulge.

Differs from *Pogonomys* (New Guinea) in having elongate incisive foramina that penetrate far into the maxilla, in having cusps strongly joined on upper and lower molars, in lacking cusp t7 on upper molars, in having cusp t4 strongly connected to cusp t5 in M1, in having cusp t9 nearly incorporated into much larger cusp t8 on M1 and M2, in having large and discrete anterolabial and anterolingual cusps on m1, in having an anterolabial cuspid on m2 that does not disrupt the anterior margin, in having a deep posterior margin between the articular condyle and the angular process, in having an angular process tilted to form a moderately wide internal shelf, and in having the mandibular foramen dorsal to the alveolar shelf.

Differs from *Sundamys* (peninsular southeast Asia, Sumatra, Borneo, Java) in having the posterior margin of the zygomatic arch opposite the occlusal surface of M1, in having only slightly slanted and overlapping cusps on upper molars, in lacking a small accessory labial cusp behind cusp t6 on M1 and M2, in having M2 with equal length and width, in having anterolabial cuspids and accessory labial cusplets on lower molars, in having large and discrete anterolabial and anterolingual cusps on m1, in having an anterolabial cuspid on m2 that does not disrupt the anterior margin, in having a coronoid process that does not extend dorsally beyond the articular condyle, and in having an angular process tilted to form a moderately wide internal shelf.

Differs from *Taeromys* (Sulawesi) in having the posterior margin of the zygomatic arch opposite the occlusal surface of M1, in having brachydont rather than hypsodont dentition, in having cusps strongly joined on upper and lower molars, in lacking cusp t7 on upper molars, in having cusp t3 present on M2 and M3, in having accessory labial cusplets on lower molars, in having large and discrete anterolabial and anterolingual cusps on m1, in having a coronoid process that does not extend dorsally beyond the articular condyle, and in having an angular process tilted to form a moderately wide internal shelf.

Differs from *Uromys* (New Guinea, Solomon Islands, northern Australia) in having elongate incisive foramina that penetrate far into the maxilla, in having the posterior margin of the zygomatic arch opposite the occlusal surface of M1, in having brachydont rather than hypsodont dentition, in lacking cusp t7 on upper molars, in having cusp t3 present on M2 and M3, in having anterolabial cuspids and accessory labial cusplets on lower molars, in having a deep posterior margin between the articular condyle and the angular process, and in having an angular process tilted to form a moderately wide internal shelf.

***Milimonggamys juliae* gen. et sp. nov.**

**Holotype:** LL 2014/1, right hemimandible with m1-3.

**Type locality:** Late Holocene horizon (3507–1889 BP), Liang Lawuala, Mahaniwa, East Sumba Regency, Sumba, Indonesia.

**Distribution:** Sumba.

**Other examined material:** Seven maxillaries and 17 dentaries (LL 2014/2–LL 2014/8, LL 2014/23–LL 2014/39), preserving variable numbers of remaining teeth and all wear stages.

**Etymology:** After Julie McLeod, to acknowledge her contribution to UK conservation and for encouraging Jennifer Crees to pursue her research interest in natural history.

**Diagnosis:** As for genus.

**Description:** Only known with certainty from upper and lower dentition and associated maxillaries and dentaries; non-associated cranial and postcranial elements may potentially be referable to the other large murine present in the same deposit, *Raksasamys tikusbesar* (see below). Soft tissue characteristics unknown.

Upper molars high-crowned; comprised of pairs or groups of discrete and partially united cusps. Anterior lamina of M1 strongly posteriorly recurved; middle and posterior laminae of M1 and laminae of M2 less strongly recurved, with cusps of M3 almost vertical. Molar laminae approximately transversely oriented and separated by deep clefts. M2 is the widest upper molar. M1 has five roots; the anterior root is very large and deep, the posterolabial root is transversely flattened and wide in cross-section, and the other three roots are circular in cross-section and relatively small. M2 has four roots arranged in a square; the two lingual roots are rounded in cross-section, and the two labial roots are transversely flattened in cross-section. M3 has two anterior roots with rounded cross-sections, with a slightly larger anterolingual root and slightly smaller anterolabial root, and a much larger single ovoid posterior root.

First upper molar (M1) with ovoid outline; widest opposite middle lamina. Anterior lamina consists of three strongly posteriorly curved columnar cusps, comprising a united but lobular t2+3 complex and discrete but adpressed t1; cusp t2 largest, strongly curved posteriorly from a large bulbous base; cusp t3 much smaller, adpressed and united along entire length of t2 and showing similar curvature; t2+3 complex with lobular anterior margin and transversely oriented combined occlusal surface; cusp t1 rounded, bulging lingually in dorsal profile and curving labially, isolated by t2+3 complex in unworn specimens by thin anterior cleft close to occlusal surface which is lost with increasing wear, with rounded occlusal surface slightly posterior to transverse plane of occlusal surface of t2+3 complex. Anterior accessory cusp absent. Middle lamina consists of three posteriorly curved columnar cusps, comprising a united but lobular t5+6 complex, and adpressed t4 which is separated by a thin cleft in specimens showing little wear but which becomes united to rest of lamina with increasing wear; cusp t5 largest, anteriorly rounded; cusp t6 only slightly smaller in size, with rounded base and flattened anterior surface, and transversely oriented occlusal surface, and adpressed and united along entire length of t5 but remaining defined by shallow, broad anterior grieve that runs to base of cleft defining the anterior border of middle lamina; cusp t4 large, rounded, bulging lingually in dorsal profile and curving labially, with occlusal surface slightly posterior to transverse plane of occlusal surface of t5+6 complex. Posterior lamina with cusp t7 absent and comprising two united but lobular, posteriorly curved columnar cusps separated by anterior groove, with anterior boundary defined by apical cleft, and with occlusal surface posterior to t5+6 complex; cusp t8 largest, broad and anteriorly rounded, with transversely oriented occlusal surface; cusp t9 smaller, with flattened anterior surface and transversely oriented occlusal surface. Posterior cingulum absent. Posterior margin of M1 leans against and slightly overlaps front face of anterior lamina of M2, overlapping up to 10% of the anterior of M2.

Second upper molar (M2) with trapezoidal outline; widest opposite anterior lamina. Cusp t1 columnar, rounded and slightly laterally flattened, situated at anterolingual corner of M2 and separated from rest of crown surface by deep cleft. Cusp t2 absent. Tiny cusp t3 present at anterolabial corner of M2, posterior to cusp t9 of M1; does not reach occlusal plane of other cusps on unworn specimens. Anterior lamina consists of three columnar cusps, comprising a united but lobular, posteriorly curved t5+6 complex, and adpressed t4 which is separated by a thin cleft in specimens showing little wear but which becomes united to rest of lamina with increasing wear; cusp t5 largest, anterior rounded; cusp t6 only slightly smaller in size, with rounded base and flattened anterior surface, with rounded base and flattened anterior surface, and transversely oriented occlusal surface, and adpressed and united along entire length of t5 but remaining defined by shallow, broad anterior grieve that runs to base of cleft defining the anterior border of anterior lamina behind t3; cusp t4 large, rounded, bulging lingually in dorsal profile and curving labially, with occlusal surface slightly posterior to transverse plane of occlusal surface of t5+6 complex. Posterior lamina with cusp t7 absent and comprising two united but lobular, posteriorly curved columnar cusps separated by anterior groove and anterior boundary defined by apical cleft, and with occlusal surface posterior to t5+6 complex; cusp t8 largest, broad and anteriorly rounded, with transversely oriented occlusal surface; cusp t9 smaller, with flattened anterior surface and transversely oriented occlusal surface. Posterior cingulum absent. Posterior margin of M2 leans against and slightly overlaps front face of anterior lamina of M3, overlapping up to 15% of the anterior of M3.

Third upper molar (M3) with rounded triangular outline; widest close to anterior margin. Cusp t1 columnar, rounded and slightly laterally flattened, situated at anterolateral corner of M3 and separated from rest of crown surface by deep cleft. Cusp t2 absent. Tiny cusp t3 present at anterolabial corner of M3, posterior to cusp t9 of M2; does not reach occlusal plane of other cusps on unworn specimens. Anterior lamina consists of three columnar cusps forming a united t4+5+6 complex in all examined specimens, with a single shared anteriorly curved chevronate occlusal surface with convex anterior margin and concave posterior margin; cusps t5 and t6 posteriorly curved, adpressed along entire length and indistinct from each other; cusp t4 adpressed and united along entire length of t5 but lobular and defined by anterior notch and shallow anterior groove. Posterior lamina with cusp t7 absent and comprising united but lobular t8+9 complex with a single shared occlusal surface in the same transverse plane, with cusps usually adpressed along entire length and indistinct from each other; cusp t8 largest, rounded; cusp t9 slightly shorter and narrower. Small posterior cingulum present at midline of posterior margin of M3, posterior to t8.

Anterior margin of anterior root of zygomatic arch situated far in front of anterior margin of M1; posterior margin opposite anterior M1, slightly in front of occlusal surface of anterior lamella. Zygomatic plate relatively broad, with thin anterior margin and well-defined u-shaped zygomatic notch, and thick posterior margin; masseteric fossa deeply excavated; attached anterior portion of zygomatic arch robust. Posterior margin of incisive foramina opposite posterior margin of M1, slightly behind anterior margin of M1. Palatal bridge relatively narrow. Palatal groove deep and broad. Posterior region of palate damaged in all available specimens, so morphology uncertain.

Lower molars high-crowned; comprised of pairs or groups of variably discrete and partially united cusps. Anteroconid of m1 and molar laminae of m1-3 separated by deep clefts, and defined across most of labial and lingual lateral molar surfaces. Occlusal plane with slight helical torsion, changing from transversely horizontal on m1 to slightly lingually inclined on m3. m1 and m2 approximately equal in width. m1 with a large ovoid or triangular anterior root, smaller rounded mediolateral and mediolingual roots, and a large, transversely elongate posterior root; m2 and m3 both with small rounded anterolingual root, larger obliquely oriented and flattened or ovoid anterolabial root, and very large transversely elongate posterior root.

First lower molar (m1) with almost triangular rounded outline, widest posteriorly and narrowing anteriorly. Anteroconid narrower than two posterior lophids; comprised of discrete but adpressed columnar anterolabial and anterolingual cuspids separated by distinct notched anterior groove, and which both curve slightly posteriorly and towards the molar midline; anterolabial cuspid slightly smaller than anterolingual cuspid; anterocentral cuspid absent. Anterior lamina chevronate, wider than posterior lamina; made up of slightly larger posterolabially situated elongate protoconid and smaller anterolingually situated columnar metaconid with broader base and narrower apex, which both curve slightly medially and are adpressed at the molar midline. Posterior lamina chevronate, made up of slightly larger posterolabially situated elongate hypoconid and posterolingually situated elongate entoconid, which are adpressed at the molar midline to form a single occlusal surface. Small anterolabial cusplet occasionally present (observed in 1 out of 19 specimens); larger posterolabial cusplet consistently present; small posterolingual cusplet also present on a few specimens. Large round posterior cingulid present at posterior margin of molar midline.

Second lower molar (m2) with slightly elongate square-shaped outline, widest opposite anterior lamina. Relatively long, oblique anterolabial cuspid present, separated from anterior lamina by etched groove; does not extend forward markedly to disrupt transverse anterior margin of tooth. Anterior lamina chevronate, slightly anteriorly curved, wider than posterior lamina; made up of slightly larger obliquely oriented elongate protoconid and smaller transversely oriented columnar metaconid, which are adpressed at the molar midline to form a single occlusal surface. Posterolabial cusplet present. Posterior lamina chevronate, slightly anteriorly curved; made up of slightly smaller obliquely oriented hypoconid and slightly larger obliquely oriented elongate entoconid, which are adpressed at the molar midline to form a single occlusal surface. Large round posterior cingulid present at posterior margin of molar midline.

Third lower molar (m3) with rounded outline, widest opposite anterior lamina. Anterolabial cuspid present. Anterior lamina transverse, wider than posterior lamina; made up of slightly larger protoconid and slightly smaller metaconid, which are adpressed at the molar midline to form a single occlusal surface. Posterior lamina transverse, anteriorly curved; made up of completely fused hypoconid and entoconid which form a single occlusal surface. Posterior cingulid absent.

Mandibular ramus shallow, deepening slightly anteriorly to reach a depth of about twice the height of the toothrow beneath anteroconid of m1, and becoming slightly deeper in older worn individuals; digastric process of symphysis not deep or robustly formed. Inferior masseteric ridge well-defined as raised ridge on external surface; superior masseteric ridge much more weakly expressed, low and rounded; ridges converge anteriorly to form rounded v-shape or bluntly rounded termination below m1 anteroconid. Mental foramen deep and well-defined immediately anterior to m1. Incisor alveolus terminates beneath posterior margin of m3; posterior end of incisor alveolus with distinct tubercle only on older, worn individuals, beneath anterior margin of coronoid process. Retromolar fossa and superior mandibular foramen well-formed; alveolar shelf supported posteriorly by postalveolar ridge. Coronoid process low (not extending above articular condyle), narrow and back-curved, with narrow mandibular notch. Articular condyle broad, with squared-off condylar head. Angular process large, with deeply excavated pterygoid fossa on internal surface; with shallow rounded ventral margin, giving the ventral margin of the overall mandible a gently chevronate outline; terminating in a gently angled posterior margin; does not extend posteriorly as far as articular process.

Measurements: upper toothrow length (occlusal) = 10.5 mm (10.0–11.1 mm, n=4); upper toothrow length (alveolar) = 10.9 mm (10.4–11.4 mm, n=6); M1 length = 5.2 mm (4.9–5.5 mm, n=6); M1 width = 2.9 mm (2.0–3.1 mm, n=6); M2 length = 3.3 mm (2.9–3.5 mm, n=7); M2 width = 2.9 mm (2.7–3.0 mm, n=7); M3 length = 2.8 mm (2.6–3.1 mm, n=6); M3 width = 2.4 mm (2.2–2.7 mm, n=5); lower toothrow length (occlusal) = 10.4 mm (9.7–10.8 mm, n=12); lower toothrow length (alveolar) = 10.5 mm (9.4–11.8 mm, n=13); m1 length = 4.3 mm (3.8–4.7 mm, n=16); m1 width = 2.8 mm (2.4–3.3 mm, n=16); m2 length = 3.3 mm (3.0–3.5 mm, n=15); m2 width = 2.8 mm (2.4–3.0 mm, n=15); m3 length = 3.0 mm (2.3–3.3 mm, n=13); m3 width = 2.5 mm (2.3–2.6 mm, n=12).

**Genus *Raksasamys* gen. nov.**

**Type species:** *Raksasamys tikusbesar* gen. et sp. nov.

**Etymology:** From the Indonesian word for “giant”, combined with “*mys*”, the standard suffix for mouse.

**Diagnosis:** Giant murine with very high-crowned and strongly fluted upper and lower molars with reduced crown morphology, with molar laminar tubes not entering the bony structure of the jaw and molar crown still visible laterally; upper molars with non-chevronate, thin flattened laminae, lacking posterior cingulum on all teeth, and strongly overlapping; M1 with central cusps t2, t5 and t8 similar in proportion to lingual cusps t1 and t4, and extremely reduced labial cusps t3, t6 and t9; tiny anteromedial accessory cusp sometimes present on M1; cusp t2 absent on M2 and M3; cusp t3 reduced on M1, and absent on M2 and M3; all upper molars lacking cusp t7; M1 with five roots, M2 with four roots, M3 with three roots; m1 with anterolabial and anterolingual cuspids but lacking anterocentral cuspid; anterolabial and posterolabial cusplets sometimes present on m1, and posterior cingulid sometimes present on m1 and m2, but all absent on m3; anterolabial cuspid present on m2, but very weakly expressed or absent on m3; m1 with four roots, m2 and m3 each with three roots; occlusal plane of lower molars with strong helical torsion, with shallow alveoli and roots; posterior margin of incisive foramina far in front of M1; posterior margin of anterior root of zygomatic arch opposite anterior M1; palatal bridge narrow, posterior palatal margin opposite M3; coronoid and angular processes large and well-developed.

*Raksasamys* differs from other southeast Asian and Australasian murids distributed outside the Lesser Sundas according to the following characters:

Differs from *Anisomys* (New Guinea) in having elongate incisive foramina that penetrate far into the maxilla, in having the posterior margin of the zygomatic arch opposite the occlusal surface of M1, in having hypsodont rather than brachydont dentition, in having cusps weakly connected on upper and lower molars, in having strongly slanted and overlapping cusps on upper molars, in having a reduced rather than an enlarged posterior cingulum on upper molars, in lacking cusp t7 on upper molars, in having cusp t4 separate from cusp t5 in M1, in having M2 with equal length and width, in lacking cusp t3 on M2 and M3, in having anterolabial cuspids on lower molars, in having an anterolabial cuspid on m2 that disrupts the anterior margin, in having a deep posterior margin between the articular condyle and the angular process, in having an angular process tilted to form a moderately wide internal shelf, and in having the mandibular foramen dorsal to the alveolar shelf.

Differs from *Bunomys* (Sulawesi) in having hypsodont rather than brachydont dentition, in having cusps weakly connected on upper and lower molars, in having cusp t4 separate from cusp t5 in M1, in having cusp t9 nearly incorporated into much larger cusp t8 on M1 and M2, in having an angular process tilted to form a moderately wide internal shelf, and in having the mandibular foramen dorsal to the alveolar shelf.

Differs from *Halmaheramys* (Halmahera) in having the posterior margin of the zygomatic arch opposite the occlusal surface of M1, in having hypsodont rather than brachydont dentition, in having cusps weakly connected on upper and lower molars, in having cusp t4 separate from cusp t5 in M1, in having M2 with equal length and width, in lacking accessory labial cusplets on lower molars, and in having large and discrete anterolabial and anterolingual cusps on m1.

Differs from *Hydromys* (New Guinea) in having elongate incisive foramina that penetrate far into the maxilla, in having three rather than two upper and lower molars present, in having hypsodont rather than brachydont dentition, in having cusps weakly connected on upper and lower molars, in having strongly slanted and overlapping cusps on upper molars, in lacking cusp t7 on upper molars, in having cusp t4 separate from cusp t5 in M1, in having M2 with equal length and width, in having anterolabial cuspids on lower molars, in having an anterolabial cuspid on m2 that disrupts the anterior margin, and in having the mandibular foramen dorsal to the alveolar shelf.

Differs from *Hyomys* (New Guinea) in having elongate incisive foramina that penetrate far into the maxilla, in having small upper and lower third molars, in having cusps weakly connected on upper and lower molars, in having a reduced rather than an enlarged posterior cingulum on upper molars, in lacking cusp t7 on upper molars, in having cusp t4 separate from cusp t5 in M1, in having cusp t9 nearly incorporated into much larger cusp t8 on M1 and M2, in lacking cusp t3 on M2 and M3, in lacking accessory labial cusplets on lower molars, in having a deep posterior margin between the articular condyle and the angular process, in having the mandibular foramen dorsal to the alveolar shelf, and in having a distinct incisor alveolus capsule defined by a high bulge.

Differs from *Lenothrix* (peninsular Malaysia, Borneo) in having the posterior margin of the zygomatic arch opposite the occlusal surface of M1, in having hypsodont rather than brachydont dentition, in having strongly slanted and overlapping cusps on upper molars, in having a reduced rather than an enlarged posterior cingulum on upper molars, in lacking cusp t7 on upper molars, in having cusp t4 separate from cusp t5 in M1, in lacking a small accessory labial cusp behind cusp t6 on M1 and M2, in having cusp t9 nearly incorporated into much larger cusp t8 on M1 and M2, in having M2 with equal length and width, in lacking cusp t3 on M2 and M3, in lacking accessory labial cusplets on lower molars, and in having a distinct incisor alveolus capsule defined by a high bulge.

Differs from *Leptomys* (New Guinea) in having hypsodont rather than brachydont dentition, in having cusps weakly connected on upper and lower molars, in having strongly slanted and overlapping cusps on upper molars, in lacking cusp t7 on upper molars, in having M2 with equal length and width, in having anterolabial cuspids on lower molars, in having an anterolabial cuspid on m2 that disrupts the anterior margin, in having a coronoid process that extends dorsally beyond the articular condyle, in having an angular process tilted to form a moderately wide internal shelf, and in having a distinct incisor alveolus capsule defined by a high bulge.

Differs from *Macruromys* (New Guinea) in having elongate incisive foramina that penetrate far into the maxilla, in having the posterior margin of the zygomatic arch opposite the occlusal surface of M1, in having hypsodont rather than brachydont dentition, in having cusps weakly connected on upper and lower molars, in having strongly slanted and overlapping cusps on upper molars, in lacking cusp t7 on upper molars, in having cusp t9 nearly incorporated into much larger cusp t8 on M1 and M2, in having M2 with equal length and width, in having anterolabial cuspids on lower molars, in having an anterolabial cuspid on m2 that disrupts the anterior margin, in having a deep posterior margin between the articular condyle and the angular process, in having the mandibular foramen dorsal to the alveolar shelf, and in having a distinct incisor alveolus capsule defined by a high bulge.

Differs from *Mallomys* (New Guinea) in having small upper and lower third molars, in lacking cusp t7 on upper molars, in lacking a small accessory labial cusp behind cusp t6 on M1 and M2, in having anterolabial cuspids on lower molars, in having large and discrete anterolabial and anterolingual cusps on m1, in having an anterolabial cuspid on m2 that disrupts the anterior margin, in having a deep posterior margin between the articular condyle and the angular process, in having the mandibular foramen dorsal to the alveolar shelf, and in having a distinct incisor alveolus capsule defined by a high bulge.

Differs from *Maxomys* (peninsular southeast Asia, Borneo, Sumatra, Java, Palawan, Sulawesi) in having hypsodont rather than brachydont dentition, in having cusps weakly connected on upper and lower molars, in having cusp t4 separate from cusp t5 in M1, in having M2 with equal length and width, in having anterolabial cuspids on lower molars, in having large and discrete anterolabial and anterolingual cusps on m1, in having an anterolabial cuspid on m2 that disrupts the anterior margin, in having a deep posterior margin between the articular condyle and the angular process, and in having a distinct incisor alveolus capsule defined by a high bulge.

Differs from *Parahydromys* (New Guinea) in having elongate incisive foramina that penetrate far into the maxilla, in having hypsodont rather than brachydont dentition, in having cusps weakly connected on upper and lower molars, in having strongly slanted and overlapping cusps on upper molars, in lacking cusp t7 on upper molars, in having cusp t4 separate from cusp t5 in M1, in having anterolabial cuspids on lower molars, in having an anterolabial cuspid on m2 that disrupts the anterior margin, in having an angular process tilted to form a moderately wide internal shelf, in having a distinct incisor alveolus capsule defined by a high bulge, and in having three rather than two upper and lower molars present.

Differs from *Paruromys* (Sulawesi) in having elongate incisive foramina that penetrate far into the maxilla, in having cusps weakly connected on upper and lower molars, in having strongly slanted and overlapping cusps on upper molars, in lacking cusp t7 on upper molars, in having cusp t9 nearly incorporated into much larger cusp t8 on M1 and M2, in lacking cusp t3 on M2 and M3, in having an anterolabial cuspid on m2 that disrupts the anterior margin, and in having an angular process tilted to form a moderately wide internal shelf.

Differs from *Phloeomys* (Philippines) in having elongate incisive foramina that penetrate far into the maxilla, in having cusps weakly connected on upper and lower molars, in having strongly slanted and overlapping cusps on upper molars, in lacking cusp t7 on upper molars, in having cusp t4 separate from cusp t5 in M1, in having M2 with equal length and width, in having anterolabial cuspids on lower molars, in having an anterolabial cuspid on m2 that disrupts the anterior margin, in having a coronoid process that extends dorsally beyond the articular condyle, in having a deep posterior margin between the articular condyle and the angular process, in having the mandibular foramen dorsal to the alveolar shelf, and in having a distinct incisor alveolus capsule defined by a high bulge.

Differs from *Pogonomys* (New Guinea) in having elongate incisive foramina that penetrate far into the maxilla, in having hypsodont rather than brachydont dentition, in having strongly slanted and overlapping cusps on upper molars, in lacking cusp t7 on upper molars, in having cusp t9 nearly incorporated into much larger cusp t8 on M1 and M2, in lacking cusp t3 on M2 and M3, in lacking accessory labial cusplets on lower molars, in having large and discrete anterolabial and anterolingual cusps on m1, in having a coronoid process that extends dorsally beyond the articular condyle, in having a deep posterior margin between the articular condyle and the angular process, in having an angular process tilted to form a moderately wide internal shelf, and in having the mandibular foramen dorsal to the alveolar shelf.

Differs from *Sundamys* (peninsular southeast Asia, Sumatra, Borneo, Java) in having the posterior margin of the zygomatic arch opposite the occlusal surface of M1, in having hypsodont rather than brachydont dentition, in having cusps weakly connected on upper and lower molars, in having cusp t4 separate from cusp t5 in M1, in lacking a small accessory labial cusp behind cusp t6 on M1 and M2, in having M2 with equal length and width, in lacking cusp t3 on M2 and M3, in having anterolabial cuspids on lower molars, in having large and discrete anterolabial and anterolingual cusps on m1, and in having an angular process tilted to form a moderately wide internal shelf.

Differs from *Taeromys* (Sulawesi) in having the posterior margin of the zygomatic arch opposite the occlusal surface of M1, in having strongly slanted and overlapping cusps on upper molars, in lacking cusp t7 on upper molars, in having cusp t4 separate from cusp t5 in M1, in having large and discrete anterolabial and anterolingual cusps on m1, in having an anterolabial cuspid on m2 that disrupts the anterior margin, and in having an angular process tilted to form a moderately wide internal shelf.

Differs from *Uromys* (New Guinea, Solomon Islands, northern Australia) in having elongate incisive foramina that penetrate far into the maxilla, in having the posterior margin of the zygomatic arch opposite the occlusal surface of M1, in having cusps weakly connected on upper and lower molars, in having strongly slanted and overlapping cusps on upper molars, in lacking cusp t7 on upper molars, in having cusp t4 separate from cusp t5 in M1, in having anterolabial cuspids on lower molars, in having an anterolabial cuspid on m2 that disrupts the anterior margin, in having a coronoid process that extends dorsally beyond the articular condyle, in having a deep posterior margin between the articular condyle and the angular process, and in having an angular process tilted to form a moderately wide internal shelf.

***Raksasamys tikusbesar* gen. et sp. nov.**

**Holotype:** LL 2014/9, right hemimandible with m1-3.

**Type locality:** Late Holocene horizon (3507–1889 BP), Liang Lawuala, Mahaniwa, East Sumba Regency, Sumba, Indonesia.

**Distribution:** Sumba.

**Other examined material:** Thirteen maxillaries, 18 dentaries, and a loose m1 (LL 2014/10–LL 2014/20, LL 2014/40–LL 2014/60), preserving variable numbers of remaining teeth and all wear stages.

**Etymology:** From the Indonesian words for “large rat”.

**Diagnosis:** As for genus.

**Description:** Only known with certainty from upper and lower dentition and associated maxillaries and dentaries; non-associated cranial and postcranial elements may potentially be referable to the other large murine present in the same deposit, *Milimonggamys juliae* (see above). Soft tissue characteristics unknown.

Upper molars extremely high-crowned; comprised of pairs or groups of variably discrete and partially united fluted cusps. M1 strongly posteriorly recurved; posterior molars less strongly recurved, with cusps of M3 almost vertical. Molar laminae transversely oriented, thin and sheet-like, and separated by deep clefts, defined on labial and lingual lateral surfaces of molars almost to the crown base. M2 is the widest upper molar. M1 has five roots; the anterior root is very large, rounded and shallow, the mediolabial root is shallow and rounded in cross-section, and the other three roots are deeper and ovoid in cross-section. M2 has four roots arranged in a square; the two lingual roots are rounded in cross-section, and the two labial roots are ovoid and laterally elongate in cross-section. M3 has two anterior roots, with the anterolabial root rounded in cross-section and the anterolingual root rounded or laterally elongate in cross-section, and a much larger single ovoid posterior root.

First upper molar (M1) with ovoid outline; widest opposite middle lamina. Anterior lamina consists of three columnar cusps, comprising a united but lobular t2+3 complex and discrete but adpressed t1; cusp t2 largest, strongly curved posteriorly and slightly lingually; cusp t3 much smaller, adpressed and united along entire length of t2 and showing similar curvature, but remaining defined by shallow anterior groove that runs to the crown base; cusp t1 short but broad, isolated from t2+3 complex by thin cleft but with occlusal surface in same transverse plane. Additional tiny anteromedial accessory cusp usually absent, but sometimes expressed as a faint, low bump which disrupts the outline of the enamel column of the anterior lamina in lateral profile, and occasionally present as a more defined, very small distinct structure close to the crown base. Middle lamina consists of three columnar cusps, comprising a united but lobular t5+6 complex, and adpressed t4 which is separated by a thin cleft in specimens showing little wear but which becomes united to rest of lamina with increasing wear; cusp t5 largest, short and curved posteriorly, with broader base and narrower apex; cusp t6 only slightly smaller in size, elongate and curved posterolingually, with broader base and narrower apex, and adpressed and united along entire length of t5 but remaining defined by distinct anterior groove that runs to base of cleft defining the anterior border of middle lamina; cusp t4 short but broad, curving labially, with occlusal surface in same transverse plane. Posterior lamina with cusp t7 absent and comprising two united but lobular columnar cusps with anterior boundary defined by apical cleft, and with occlusal surface posterior to t5+6 complex; cusp t8 largest, broad and curved posterolabially; cusp t9 smaller, curved posterolingually. Posterior cingulum absent. Posterior margin of M1 leans against and overlaps front face of anterior lamina of M2, overlapping approximately the anterior third of M2.

Second upper molar (M2) with trapezoidal outline; widest opposite anterior lamina. Cusp t1 columnar, curving labially, situated at anterolingual corner of M2 and separated from rest of crown surface by deep cleft. Cusps t2 and t3 absent. Anterior lamina consists of three columnar cusps, comprising a united but lobular t5+6 complex, and adpressed t4 which is separated by a thin cleft in specimens showing little wear but which becomes united to rest of lamina with increasing wear; cusp t5 largest, short and slightly curved posteriorly, with broader base and narrower apex; cusp t6 only slightly smaller in size, slightly curved posterolingually, with broader base and narrower apex, and adpressed and united along entire length of t5 but remaining defined by distinct anterior groove that runs to base of anterior border; cusp t4 short but broad, curving labially, with occlusal surface in same transverse plane. Posterior lamina with cusp t7 absent and comprising two united but lobular columnar cusps with anterior boundary defined by apical cleft, and with occlusal surface posterior to t5+6 complex; cusp t8 largest, broad and oriented posterolabially; cusp t9 smaller, oriented posterolingually, reduced to a small labial appendix of t8 with wear. Posterior cingulum absent. Posterior margin of M2 leans against and overlaps front face of anterior lamina of M3, overlapping approximately the anterior third of M3.

Third upper molar (M3) with square outline; widest opposite anterior lamina. Cusp t1 columnar, slightly curved labially, situated at anterolateral corner of M3, separated posteriorly from anterior lamina by deep cleft but adpressed and largely united anteriorly and medially except in very unworn specimens. Cusps t2 and t3 absent. Anterior lamina consists of three columnar cusps forming a united t4+5+6 complex with a single shared occlusal surface in the same transverse plane; cusp t5 largest, rounded; cusp t6 short and narrow, adpressed along entire length of t5 and indistinct; cusp t4 short and narrow, adpressed and united along entire length of t5 but lobular and defined by anterior notch and shallow anterior groove. Posterior lamina consists of united but lobular shared occlusal surface, apparently composed of united t8+9 complex in the same transverse plane; cusp t8 largest, rounded; cusp t9 shorter and narrower, adpressed along entire length of t8 but defined by anterolateral notch and shallow groove.

Anterior margin of anterior root of zygomatic arch situated far in front of anterior margin of M1; posterior margin opposite middle lamella of M1. Zygomatic plate broad, with thin anterior margin and well-defined, shallow v-shaped zygomatic notch, and thick posterior margin; masseteric fossa deeply excavated; attached anterior portion of zygomatic arch very robust. Posterior margin of incisive foramina markedly anterior to M1, approximately halfway along zygomatic plate. Palatal bridge narrow. Palatal groove deep and broad, running along inner length of entire toothrow. Posterior palatal foramen opposite front of M3. Posterior palatal margin opposite middle of M3.

Lower molars extremely high-crowned; comprised of pairs or groups of variably discrete and partially united fluted cusps. Molar laminae separated by deep clefts, with m1 anteroconid and other laminae defined across most of lateral molar surfaces. Occlusal plane with strong helical torsion, changing from transversely horizontal on m1 to lingually inclined. m2 is the widest lower molar. m1 with a large rounded anterior root, smaller rounded mediolateral and mediolingual roots, and a large, transversely elongate posterior root; m2 and m3 both with rounded anterolingual and anterolabial roots, an accessory labial rootlet situated posterior to the anterolabial root, and a larger, rounded ovoid posterior root; alveoli and roots shallow in most individuals, only becoming deeply expressed in old, worn individuals.

First lower molar (m1) with almost triangular rounded outline, widest posteriorly and narrowing anteriorly. Anteroconid and anterior lamina non-chevronate, posterior lamina chevronate, all anterolingually oriented by c.40 degrees. Anteroconid narrower and shorter than two posterior lophids; comprised of discrete but adpressed smaller anterolabial cuspid and larger anterolingual cuspid separated by relatively shallow anterior groove; anterocentral cuspid absent. Anterior lamina wider than posterior lamina; made up of smaller, posterolabially situated protoconid and larger, elongate, anterolingually situated columnar metaconid with broader base and narrower apex, which both curve inwards towards the molar midline, adpressing at the level of the occlusal plane but separated closer to the crown base in unworn specimens. Posterior lamina made up of rounded triangular, posterolabially situated hypoconid with broader base and narrower apex, and narrower, anterolingually situated and anterolabially curving columnar entoconid, which are discrete but adpressed along their length, remaining distinct in specimens with little wear and only merging to form a single occlusal surface with moderate wear; defined posteriorly by deep, narrow cleft. Tiny anterolabial cusplet, posterolabial cusplet and posterior cingulid absent from most specimens, but present in a few examined specimens; intraspecific variation in these structures is also seen within both species of *Papagomys* (Zijlstra et al. 2008).

Second lower molar (m2) with approximately square-shaped outline, widest opposite anterior lamina. Laminae chevronate; posterior lamina more strongly chevronate than anterior lamina. Anterolabial cuspid present; extends forward to disrupt the tooth’s transverse anterior margin. Anterior lamina almost transversely oriented; made up of similar-sized columnar protoconid and metaconid, which both curve inwards towards the molar midline, adpressing at the level of the occlusal plane but separated closer to the crown base in unworn specimens. Posterior lamina slightly anterolingually oriented; made up of similar-sized, forward-curving columnar hypoconid and entoconid, which are discrete but adpressed along their length, remaining distinct in specimens with little wear and only merge to form a single occlusal surface with moderate wear; defined posteriorly by deep, narrow cleft. Posterolabial cusplet absent; posterior cingulid usually absent, but present on a few examined specimens.

Third lower molar (m3) with rounded triangular outline, widest opposite anterior lamina; slightly longer than m2. Anterolabial cuspid either absent or expressed as tiny enamel ridge on corner of protoconid. Anterior lamina transverse; made up of completely fused protoconid and metaconid forming a single occlusal surface. Posterior lamina transverse, slightly anterolingually oriented; narrower and longer than anterior lamina; made up of completely fused hypoconid and entoconid forming a single occlusal surface. Posterior cingulid absent.

Mandibular ramus shallow and strap-like, deepening slightly anteriorly to reach a depth of about 1.5 times the height of the toothrow beneath anteroconid of m1; digastric process of symphysis not deep or robustly formed. Superior and inferior masseteric ridges well-defined on external surface, raised up as low ridges which converge anteriorly to form bluntly rounded, “squared-off” termination below m1 anteroconid. Mental foramen deep and well-defined immediately anterior to m1. Incisor alveolus terminates behind posterior margin of m3; posterior end of incisor alveolus with distinct tubercle on external surface beneath anterior margin of mandibular notch. Alveolar depression very shallow, and with strong helical torsion. Retromolar fossa and superior mandibular foramen well-formed; alveolar shelf supported posteriorly by postalveolar ridge, which carries a prominent groove on medial surface beneath the retromolar fossa, and is notched beneath the superior mandibular foramen. Coronoid process back-curved with narrow mandibular notch; rises sharply beside m3 and higher than articular condyle, producing a tall ascending ramus and giving the overall mandible a slightly “stubby” appearance. Articular condyle broad, with squared-off condylar head. Angular process large, with deeply excavated pterygoid fossa on internal surface, and as long as articular condyle; with shallow rounded ventral margin, giving the ventral margin of the overall mandible a gently chevronate outline; terminating in an angled posterior margin.

Measurements: upper toothrow length (occlusal) = 13.2 mm (12.3–14.1 mm, n=3); upper toothrow length (alveolar) = 14.7 mm (12.6–16.0 mm, n=9); M1 length = 7.3 mm (6.9–7.7 mm, n=7); M1 width = 4.2 mm (3.9–4.5 mm, n=7); M2 length = 4.8 mm (4.5–5.3 mm, n=6); M2 width = 3.9 mm (3.8–4.1 mm, n=6); M3 length = 3.7 mm (3.6–3.9 mm, n=3); M3 width = 3.1 mm (3.0–3.3 mm, n=3); lower toothrow length (occlusal) = 13.1 mm (12.2–14.0 mm, n=9); lower toothrow length (alveolar) = 13.5 mm (12.6–14.3 mm, n=14); m1 length = 4.8 mm (4.2–5.5 mm, n=18); m1 width = 3.4 mm (2.9–3.9 mm, n=18); m2 length = 3.8 mm (3.4–4.2 mm, n=16); m2 width = 3.7 mm (3.3–4.2 mm, n=16); m3 length = 4.1 mm (3.7–4.6 mm, n=9); m3 width = 3.7 mm (3.3–4.1 mm, n=9).

**Text S3: Phylogenetic placement of *Milimonggamys* and *Raksasamys***

In order to investigate the phylogenetic placement of *Milimonggamys* and *Raksasamys*, we described and coded craniodental characters from the material reported in this study, together with data for other endemic murids from the Lesser Sunda Islands (*Hooijeromys*, *Komodomys*, *Papagomys*, *Paulamys*, *Rattus hainaldi*, *Rattus timorensis*), and other southeast Asian and Australasian Rattini (*Bunomys*, *Halmaheramys*, *Lenothrix*, *Maxomys*, *Paruromys*, *Sundamys*, *Taeromys*), Hydromyini (*Anisomys*, *Hydromys*, *Hyomys*, *Leptomys*, *Macruromys*, *Mallomys*, *Parahydromys*, *Pogonomys*, *Uromys*), and Phloeomyini (*Phloeomys*) (see Fabre et al. 2013; Pagès et al. 2016). The extinct endemic Lesser Sunda murid genera *Coryphomys* and *Spelaeomys* were excluded from phylogenetic analysis, as these taxa are clearly morphologically distinct and evolutionarily distant from other murids from Flores and Sumba (Musser 1981; Aplin and Helgen 2010). This wider biogeographic murid sample includes taxa from Wallacea (Halmahera, Sulawesi: *Bunomys*, *Halmaheramys*, *Paruromys*, *Taeromys*), Sahul and neighbouring islands (New Guinea, Australia, the Solomon Islands: *Anisomys*, *Hydromys*, *Hyomys*, *Leptomys*, *Macruromys*, *Mallomys*, *Parahydromys*, *Pogonomys*, *Uromys*), the Philippine biogeographic region (*Phloeomys*), and the Sunda Shelf (peninsular southeast Asia, Sumatra, Borneo, Java, Palawan: *Lenothrix*, *Maxomys*, *Sundamys*).

**Methods:** Our analysis used four sources for the majority of coded craniodental characters: characters 1-6 correspond to characters defined by Aplin and Helgen (2010: Table 4); characters 7-16 correspond to characters defined by Musser (1981); characters 17-19 are characters derived from descriptions of the new taxa in this study; and characters 20-22 are based upon descriptions from Kitchener et al. (1991b). We further defined characters 23-25.

We excluded characters from previous murid phylogenetic studies that could not be coded for *Milimongamys* and *Raksasamys*. We also excluded Aplin and Helgen (2010)’s character referring to anteroconid elaboration, due to this character offering only ambiguous phylogenetic information for *Coryphomys* and by extension for other murids, and due to the multiple apparent derivations of this structure (Aplin and Helgen 2010: p.54). We used published data from Musser (1981) to code for *Hooijeromys*, *Komodomys*, *Lenothrix*, *Maxomys*, *Papagomys* and *Paulamys*; from Musser and Newcomb (1983) for *Sundamys*; from Kitchener et al. (1991b) for *Bunomys*, *Komodomys*, *Lenothrix*, *Maxomys* and *Paulamys*; from Kitchener et al. (1991c) for *Rattus hainaldi*; from Kitchener et al. (1991a) for *Rattus timorensis*; from Aplin and Helgen (2010) for *Lenothrix* and *Phloeomys* (as Phloeomyini); and from Fabre et al. (2013) for *Halmaheramys*. We collected data for *Anisomys*, *Bunomys*, *Hydromys*, *Hyomys*, *Leptomys*, *Macruromys*, *Mallomys*, *Parahydromys*, *Paruromys*, *Pogonomys*, *Taeromys* and *Uromys* from specimens in the collections of the Natural History Museum, London.

Characters and states are defined as follows; corresponding character numbers from Musser (1981) are given in square parentheses for characters 7-16:

**1.** Cusp t7 on upper molars: (0) absent; (1) present.

**2.** Anterolabial cuspids on lower molars: (0) present; (1) absent (see Helgen and Alpin 2010 for polarity discussion).

**3.** Accessory labial cusplets on lower molars: (0) present; (1) absent.

**4.** Posterior molar size: (0) large; (1) small.

**5.** Status of posterior cingulum on upper molars: (0) not enlarged; (1) enlarged.

**6.** Length of incisive foramina: (0) elongate, penetrating far into maxilla; (1) short, narrow.

**7.** [9] Palatal bridge: (0) “posterior rim of the palatal bridge is situated before the back margins of the third upper molars, even with them, or only slightly beyond them”; (1) “posterior part of the palatal bridge extends way beyond the third molars to form a wide platform behind the molar rows”.

**8.** [10] Mesopterygoid fossa: (0) “fossa is nearly as wide as the back part of the palatal bridge; its walls are breached by thin, short sphenopalatine vacuities or slits”; (1) “fossa is one-third to one-half the width of the palatal bridge; the sphenopalatine vacuities are huge so that the presphenoid and anterior part of the basisphenoid appear suspended in air”.

**9.** [16] Height of cusps: (0) “molars have low to moderately high cusps, but definitely brachyodont”; (1) “molars have high cusps, clearly hypsodont”.

**10.** [18] Overlap of molars: (0) “cusps on the upper molars are only slightly slanted and there is little or no overlap among the three teeth in a toothrow, each essentially abutting against the other”; (1) “cusps slant conspicuously back, so that the first molar overlaps the second, and the second overlaps the third”.

**11.** [22] Cusp t9: (0) “large and discrete on each first and second upper molar”; (1) “nearly incorporated into the much larger cusp t8 and inconspicuous after wear”.

**12.** [23] Cusp t3: (0) “in all or most of the samples, cusp t3 is present on the second and often on the third upper molars” (1) “absent from the second and third molars in all or most specimens”.

**13.** [24] Cusp behind t6 (C-t6): (0) “a small accessory labial cusp is not present behind cusp t6 on the first or second upper molars of most specimens”; (1) “a small accessory cusp sits directly behind cusp t6 on each first upper molar and sometimes on each second molar in most or all specimens; as each tooth wears down, the cusp merges with the back of cusp t6 to form a crest”.

**14.** [25] Relationship between cusps t4 and t5: (0) “cusp t4 is separate from cusp t5 in the first upper molars of juveniles and adults”; (1) “cusp t4 strongly connected to cusp t5, sometimes weakly connected only in juveniles”.

**15.** [27] Union of cusps: (0) “cusps on the upper and lower molars are weakly connected so the occlusal patterns appear strongly cuspidate”; (1) “all or most cusps are strongly joined, some merged to the point where they nearly lose their identities”.

**16.** [28] Anterolabial and anterolingual cusps on m1: (0) “large and discrete, forming a lamina nearly as wide as the rest of the tooth”; (1) “smaller, pressed against each other to form a lamina narrower than the rest of the tooth; in young rats they may be separate but soon merge into an oblong lamina”.

**17.** M2 length and width: (0) equal; (1) not equal.

**18.** Anterior labial cuspid on m2: (0) disrupts margin; (1) does not disrupt margin.

**19.** Position of anterior edge of posterior margin of zygomatic arch when viewed ventrally: (0) opposite occlusal surface of M1 (behind anterior margin of toothrow); (1) anterior to occlusal surface of M1.

**20.** Height of coronoid process: (0) does not extend dorsally beyond articular condyle; (1) extends dorsally beyond articular condyle.

**21.** Posterior margin between articular condyle and angular process: (0) deep; (1) shallow.

**22.** Ventral surface of angular process: (0) only slightly tilted into a shelf; (1) tilted to form moderately wide internal shelf.

**23.** Mandibular foramen placement: (0) within alveolar shelf; (1) dorsal to alveolar shelf.

**24.** Incisor alveolus capsule: (0) no, or very low, tubercle at posterior end of incisor alveolus on labial surface of ramus; (1) distinct, high bulge on external surface of ramus at base of coronoid process.

**25.** Number of molars: (0) three; (1) two.

We combined these data with molecular data for four gene matrices used in Fabre et al. (2013)—one mitochondrial gene (cytochrome *b*); two nuclear genes (growth hormone receptor exon 10, *GHR*; interphotoreceptor retinoid binding protein exon 1, *IRPB*); and a nuclear plus mitochondrial supermatrix—to run a Total Evidence analysis. This resulted in a matrix of 25 taxa and 5966 characters (25 morphological and 5941 DNA characters) (table S3). Eight taxa (*Hooijeromys, Komodomys*, *Milimonggamys*, *Papagomys, Paulamys*, *Raksasamys*, *Rattus hainaldi, R. timorensis*) had no DNA data. We ran a combined analysis in MrBayes using an Mk model for the morphological data and a GTR model for the molecular data, both with four discrete rate categories drawn from a gamma distribution. We ran chains for 5M generations on two runs, with four chains each (one hot, three cold) with a stop rule when the chains converged (ASDS < 0.01). We then generated a majority consensus tree from the saved trees (representing clades appearing in >50% of the iterations) for the combined data, for molecular data only, and for morphological data only (figure S6). We also generated a parsimony-based tree in PAUP through a heuristic search with 100 random addition sequences.

**Results:** The majority consensus tree using molecular-only data was able to resolve two major clades, comprising the Rattini and the Hydromyini, which form a trichotomy with *Phloeomys* (Phloeomyini) (figure S6). Sampled representatives of the Rattini ((((((*Taeromys*, *Paruromys*), *Bunomys*), *Halmaheramys*), *Sundamys*), *Lenothrix*), *Maxomys*) all show the same relationships as in the analysis of Fabre et al. (2013), as we would expect (although this previous analysis did not include *Lenothrix*). The Hydromyini (((((*Hydromys*, *Parahydromys*), *Leptomys*), *Uromys*), *Mallomys*), *Anisomys*) is also relatively well defined, although the relationship between *Hyomys*, *Macruromys* and *Pogonomys* is unresolved within this clade.

The combined evidence tree also returned distinct Rattini and Hydromyini clades in a trichotomy with *Phloeomys*, and returned a topology identical to the DNA-only tree for the Hydromyini (figure S6). *Milimonggamys*, *Raksasamys*, and all of the endemic Lesser Sunda murids were placed within the Rattini, although the relationships of *Milimonggamys* and *Raksasamys* to other Rattini are unresolved due to polytomy within this clade.

The majority consensus trees for the Bayesian and parsimony analyses of morphology-only data showed varying levels of resolution, with the Bayesian tree being largely unresolved (figure S6). Neither of the trees was able to return monophyletic Rattini or Hydromyini clades, with taxa from both of these molecular clades distributed across the morphology-only trees. Previous workers (e.g. Aplin and Helgen 2010) have recommended against investigating southeast Asian and Australasian murid phylogenetic relationships using morphological data alone due to the large amount of homoplasy evident in Rattini and Hydromyini phenotypes. The failure to resolve Rattini and Hydromyini clades and the generally poor resolution of both the Bayesian and parsimony analyses of morphology-only data is probably due to morphological data adding noise to the analyses, and almost all of the taxa for which only morphological data were available (*Hooijeromys*, *Komodomys*, *Lenothrix, Milimonggamys*, *Papagomys, Raksasamys*, *Rattus hainaldi, Rattus timorensis*) grouped as a polytomy in the combined tree.

However, both of the majority consensus morphology-only trees were able to provide some resolution on the relationships of *Milimonggamys*, *Raksasamys*, and other Lesser Sunda endemic murids even in the absence of molecular data. Two sister-taxon relationships, (1) between *Raksasamys* and the Floresian endemic *Papagomys*, and (2) between the Floresian endemic *Paulamys* and the Sunda Shelf Rattini genus *Maxomys*, were returned from both the Bayesian and parsimony analyses. However, the *Paulamys* + *Maxomys* clade is nested unexpectedly within a clade otherwise composed of hydromyine genera, and explains why both genera are placed outside Rattini in the combined evidence tree. Parsimony analysis also identified a further sister-taxon relationship, (3) between the Floresian endemic *Komodomys* and the New Guinea genus *Pogonomys*. A further clade comprising two hydromyine genera, (4) *Hydromys* + *Parahydromys*, is also consistent across both trees.

**Table S1.** Caves investigated for Quaternary fossils during palaeontological fieldwork on Sumba in June-July 2014.

| **Cave** | **Latitude (S)** | **Longitude (E)** | **Elevation (m)** | **Date of visit** |
| --- | --- | --- | --- | --- |
| Liang Lawuala | 10.032 | 120.168 | 833 | 23-25 June 2014 |
| Liang Minabuti | 10.029 | 120.171 | 830 | 23, 26 June 2014 |
| Liang Lawuala II | 10.034 | 120.168 | 925 | 23 June 2014 |
| Liang Lawuala III | 10.032 | 120.168 | 833 | 24 June 2014 |
| Liang Tiring Katehu | 10.039 | 120.167 | 782 | 24 June 2014 |
| Liang Galla Awang | 10.031 | 120.170 | 780 | 24 June 2014 |
| Liang Galla Awang II | 10.030 | 120.169 | 974 | 24 June 2014 |
| Unnamed cave | 10.151 | 120.759 | 21 | 1 July 2014 |
| Liang Wakatana | 10.118 | 120.724 | 83 | 1 July 2014 |
| Liang Wakatana II | 10.118 | 120.725 | 87 | 1 July 2014 |
| Liang Kaliawar | 10.046 | 120.751 | 141 | 1 July 2014 |
| Liang Kahembi | 9.967 | 120.713 | 130 | 1 July 2014 |
| Unnamed cave | 10.045 | 120.750 | 133 | 2 July 2014 |
| Liang Lakamaru | 10.039 | 120.751 | 116 | 2 July 2014 |
| Unnamed cave | 10.034 | 120.747 | 155 | 2 July 2014 |
| Waikello Sawah | 9.596 | 119.340 | 417 | 5 July 2014 |
| Unnamed cave | 9.594 | 119.346 | 449 | 5 July 2014 |
| Unnamed cave | 9.612 | 119.371 | 390 | 5 July 2014 |
| Liang Wanno Gaspar | 9.610 | 119.374 | 428 | 5 July 2014 |
| Liang Roma Weliang | 9.608 | 119.370 | 433 | 5 July 2014 |
| Liang Kanabu Wulang | 9.664 | 119.859 | 458 | 6 July 2014 |

**Table S2.** Upper and lower molar toothrow crown lengths for endemic murids from the eastern Lesser Sundas, and associated body mass estimates calculated using the regression equation for <5 kg muroid lower toothrow length against body mass in Hopkins (2008). Body mass estimate for *Hooijeromys nusatenggara* calculated using upper toothrow length, as lower toothrow measurement data are unavailable; this estimate should therefore be treated as an approximation.

| **Species** | **Island** | **Upper toothrow (mm)** | **Lower toothrow (mm)** | **Body mass (g)** | **Reference** |
| --- | --- | --- | --- | --- | --- |
| *Hooijeromys nusatenggara* | Flores | 11.5–12.4 (n=2) | — | 398.8–489.4 | Musser (1981) |
| *Komodomys rintjanus* | Flores | — | 7.0–7.6 (n=13) | 103.6–129.5 | Musser (1981) |
| *Papagomys armandvillei* | Flores | 14.1–16.0 (n=9) | 13.8–16.1 (n=12) | 654.4–994.6 | Musser (1981) |
| *Papagomys theodorverhoeveni* | Flores | — | 12.0–14.0 (n=11) | 447.7–680.5 | Musser (1981) |
| *Paulamys naso* | Flores | 7.1 (n=1) | 7.2–7.6 (n=4) | 111.8–129.5 | Musser (1981), Kitchener et al. (1991b) |
| *Rattus hainaldi* | Flores | 5.4 (n=1) | 5.5 (n=1) | 53.8 | Kitchener et al. (1991c) |
| *Spelaeomys florensis* | Flores | 13.4–14.6 (n=2) | 13.8–15.4 (n=19) | 654.4–881.5 | Musser (1981) |
| *Milimonggamys juliae* | Sumba | 10.0–11.1 (n=4) | 9.7–10.8 (n=12) | 251.2–336.3 | this study |
| *Raksasamys tikusbesar* | Sumba | 12.3–14.1 (n=3) | 12.2–14.0 (n=9) | 468.2–680.5 | this study |
| *Coryphomys buehleri* | Timor | 19.5 (n=1) | 18.8–20.1 (n=3) | 1515.4–1817.2 | Aplin & Helgen (2010) |
| *Coryphomys musseri* | Timor | 17.1 (n=1) | 17.2–18.8 (n=12) | 1190.2–1515.4 | Aplin & Helgen (2010) |
| *Rattus timorensis* | Timor | 6.9 (n=1) | 6.3 (n=1) | 77.79 | Kitchener et al. (1991a) |

**Table S3.** Morphological character matrix used in phylogenetic analyses of southeast Asian and Australasian murids.

|  | **Morphological character number** | | | | | | | | | | | | | | | | | | | | | | | | |
| --- | --- | --- | --- | --- | --- | --- | --- | --- | --- | --- | --- | --- | --- | --- | --- | --- | --- | --- | --- | --- | --- | --- | --- | --- | --- |
| **Taxon** | **1** | **2** | **3** | **4** | **5** | **6** | **7** | **8** | **9** | **10** | **11** | **12** | **13** | **14** | **15** | **16** | **17** | **18** | **19** | **20** | **21** | **22** | **23** | **24** | **25** |
| *Anisomys imitator* | 1 | 1 | 1 | 1 | 1 | 1 | 1 | 1 | 0 | 0 | 1 | 0 | 0 | 1 | 1 | 0 | 1 | 1 | 1 | 1 | 1 | 0 | 0 | 1 | 0 |
| *Bunomys chrysocomus* | 0 | 0 | ? | 1 | 0 | 0 | 0 | 0 | 0 | 1 | 0 | 1 | 0 | 1 | 1 | 0 | 0 | ? | 0 | 1 | 0 | 0 | 0 | 1 | 0 |
| *Halmaheramys bokimekot* | 0 | 0 | 0 | 1 | 0 | 0 | 0 | ? | 0 | 1 | 1 | 1 | 0 | 1 | 1 | 1 | 1 | 0 | 1 | 1 | 0 | ? | 1 | 1 | 0 |
| *Hooijeromys nusatenggara* | 0 | 0 | 1 | 1 | 0 | 0 | ? | ? | 0 | 1 | 1 | 0 | 0 | 1 | 0 | 1 | 1 | ? | ? | ? | ? | ? | ? | ? | 0 |
| *Hydromys chrysogaster* | 1 | 1 | ? | 1 | 0 | 1 | 1 | 1 | 0 | 0 | 1 | ? | ? | 1 | 1 | 0 | 1 | 1 | 0 | 1 | 0 | 1 | 0 | 1 | 1 |
| *Hyomys goliath* | 1 | 0 | 0 | 0 | 1 | 1 | 0 | 1 | 1 | 1 | 0 | 0 | 0 | 1 | 1 | 0 | 0 | 0 | 0 | 1 | 1 | 1 | 0 | 0 | 0 |
| *Komodomys rintjanus* | 0 | 0 | 1 | 1 | 0 | 0 | 0 | 1 | 0 | 1 | 0 | 0 | 0 | 1 | 0 | 1 | 0 | 1 | 0 | 0 | 0 | ? | 0 | ? | 0 |
| *Lenothrix canus* | 1 | 0 | 0 | 1 | 1 | 0 | 0 | 0 | 0 | 0 | 0 | 0 | 1 | 1 | 0 | 0 | 1 | 0 | 1 | 1 | 0 | 1 | 1 | 0 | 0 |
| *Leptomys elegans* | 1 | 1 | ? | 1 | 0 | 0 | 1 | 1 | 0 | 0 | 1 | 1 | 0 | 0 | 1 | 0 | 1 | 1 | 0 | 0 | 0 | 0 | ? | 0 | 0 |
| *Macruromys major* | 1 | 1 | ? | 1 | 0 | 1 | 0 | 1 | 0 | 0 | 0 | 1 | 0 | 0 | 1 | 0 | 1 | 1 | 1 | 1 | 1 | 1 | 0 | 0 | 0 |
| *Mallomys rothschildi* | 1 | 1 | 1 | 0 | 0 | 0 | 0 | 1 | 1 | 1 | 1 | 1 | 1 | 0 | 0 | 1 | 0 | 1 | 0 | 1 | 1 | 1 | 0 | 0 | 0 |
| *Maxomys whiteheadi* | 0 | 1 | 1 | 1 | 0 | 0 | 0 | 0 | 0 | 1 | 1 | 1 | 0 | 1 | 1 | 1 | 1 | 1 | 0 | 1 | 1 | 1 | 1 | 0 | 0 |
| *Milimonggamys juliae* | 0 | 0 | 0 | 1 | 0 | 0 | ? | ? | 0 | 0 | 1 | 0 | 0 | 1 | 1 | 0 | 0 | 1 | 0 | 0 | 0 | 1 | 1 | 1 | 0 |
| *Papagomys armandvillei* | 0 | 0 | 0 | 1 | 0 | 0 | 0 | 0 | 1 | 1 | 1 | 0 | 0 | 0 | 0 | 0 | 1 | 0 | 0 | 1 | 0 | 1 | 1 | 1 | 0 |
| *Parahydromys asper* | 1 | 1 | 1 | 1 | 0 | 1 | 1 | 1 | 0 | 0 | 1 | ? | ? | 1 | 1 | 0 | 0 | 1 | 0 | 1 | 0 | 0 | 1 | 0 | 1 |
| *Paruromys dominator* | 1 | 0 | ? | 1 | 0 | 1 | 1 | 1 | 1 | 0 | 0 | 0 | 0 | 0 | 1 | 0 | 0 | 1 | 0 | 1 | 0 | 0 | 1 | 1 | 0 |
| *Paulamys naso* | 0 | 1 | 1 | 1 | 0 | 1 | 0 | 0 | 0 | 1 | 1 | 1 | 0 | 1 | 1 | 0 | 1 | 1 | 0 | 1 | ? | 1 | 1 | ? | 0 |
| *Phloeomys cumingi* | 1 | 1 | 1 | 1 | 0 | 1 | 1 | 1 | 1 | 0 | 1 | 1 | 0 | 1 | 1 | 0 | 1 | 1 | 0 | 0 | 1 | 1 | 0 | 0 | 0 |
| *Pogonomys loriae* | 1 | 0 | 0 | 1 | 0 | 1 | 0 | 1 | 0 | 0 | 0 | 0 | 0 | 0 | 0 | 1 | 0 | 0 | 0 | 0 | 1 | 0 | 0 | 1 | 0 |
| *Raksasamys tikusbesar* | 0 | 0 | 1 | 1 | 0 | 0 | ? | ? | 1 | 1 | 1 | 1 | 0 | 0 | 0 | 0 | 0 | 0 | 0 | 1 | 0 | 1 | 1 | 1 | 0 |
| *Rattus hainaldi* | 1 | 0 | 0 | 1 | 0 | 0 | 1 | ? | 0 | 1 | 0 | 1 | 0 | 0 | 0 | 1 | 1 | 0 | 0 | 1 | 0 | ? | ? | ? | 0 |
| *Rattus timorensis* | 0 | ? | ? | 1 | ? | 0 | 1 | ? | 0 | 0 | ? | 1 | 0 | 1 | ? | ? | 0 | ? | 1 | 1 | 0 | 1 | ? | 1 | 0 |
| *Sundamys infraluteus* | 0 | 1 | 1 | 1 | 0 | 0 | 1 | ? | 0 | 1 | 1 | 0 | 1 | 1 | 1 | 1 | 1 | 0 | 1 | 1 | 0 | 0 | ? | 1 | 0 |
| *Taeromys celebensis* | 1 | 0 | 1 | 1 | 0 | 0 | 1 | 1 | 1 | 0 | 1 | 1 | 0 | 1 | 0 | 1 | 0 | 1 | 1 | 1 | 0 | 0 | 1 | 1 | 0 |
| *Uromys caudimaculatus* | 1 | 1 | 1 | 1 | 0 | 1 | 1 | 1 | 1 | 0 | 1 | 1 | ? | 1 | 1 | 0 | 0 | 1 | 1 | 0 | 1 | 0 | 1 | 1 | 0 |

**Table S4.** Maxillary tooth measurements for *Varanus hooijeri* specimens from Flores and Sumba, and compared to measurements from the extant bunodont varanid *V. olivaceus*. Measurements in mm; data for Flores specimens and convention for numbering teeth taken from Brongersma (1958); data for *V. olivaceus* from Auffenberg (1988).

| **Measurement** | **Flores specimens** | **LL 2014/21** | **LL 2014/22** | ***V. olivaceus*** |
| --- | --- | --- | --- | --- |
| 4th tooth: length | 2.8–3.1 (n=2) | 1.2 | — | — |
| 4th tooth: width | 2.3–2.5 (n=2) | 1.4 | — | — |
| 5th tooth: length | 2.9 (n=1) | 1.7 | 2.4 | — |
| 5th tooth: width | 2.8 (n=1) | 1.9 | 3.0 | — |
| 6th tooth: length | 3.5–4.2 (n=2) | 2.3 | 3.1 | — |
| 6th tooth: width | 4.6–6.0 (n=2) | 2.7 | 4.0 | — |
| 7th tooth: length | 3.8–5.3 (n=2) | 3.6 | 3.5 | — |
| 7th tooth: width | 4.7–6.3 (n=2) | 3.1 | 4.3 | — |
| 8th tooth: length | — | — | 2.0 | — |
| 8th tooth: width | — | — | 2.6 | — |
| Maxillary height | — | 7.0 | 9.4 | — |
| Maxillary length | 27.5+ – 29.9+ (n=2) | 15.4+ | 27.3 | 44.8–57.2 (n=24) |
| Maxillary height×100/length | — | <45.5 | 34.4 | 26.0–41.5 (n=24) |

**Table S5.** Uranium-series isotope data from line analyses of *Stegodon* molar and 126 ka-old coral standard (MK16). Legend: 2SE = 2 standard errors on the mean calculated using Iolite™.

| Line |  | Dimensionless distance | U (ppm) | 2SE | Th (ppm) | 2SE | (^232^Th/^238^U) | 2SE | (^234^U/^238^U) | 2SE | (^230^Th/^238^U) | 2SE |
| --- | --- | --- | --- | --- | --- | --- | --- | --- | --- | --- | --- | --- |
| 1 | Dentine | -1.00 | 92.40 | 2.70 | 0.0327 | 0.0021 | 0.000118 | 0.000004 | 1.19 | 0.01 | 1.58 | 0.03 |
| 2 | Dentine | -0.88 | 65.70 | 1.80 | 0.0162 | 0.0014 | 0.000082 | 0.000006 | 1.25 | 0.01 | 1.73 | 0.01 |
| 3 | Dentine | -0.76 | 54.80 | 1.10 | 0.00932 | 0.00049 | 0.000056 | 0.000003 | 1.30 | 0.00 | 1.79 | 0.02 |
| 4 | Dentine | -0.65 | 43.60 | 1.40 | 0.00486 | 0.00024 | 0.000036 | 0.000002 | 1.32 | 0.01 | 1.86 | 0.01 |
| 5 | Dentine | -0.54 | 36.70 | 1.60 | 0.00362 | 0.00019 | 0.000032 | 0.000002 | 1.35 | 0.01 | 1.83 | 0.02 |
| 6 | Dentine | -0.43 | 42.00 | 2.90 | 0.0069 | 0.0012 | 0.000057 | 0.000012 | 1.32 | 0.01 | 1.76 | 0.03 |
| 7 | Dark enamel | -0.37 | 1.19 | 0.12 | 0.0002 | 0.00019 | 0.000061 | 0.000062 | 1.05 | 0.02 | 0.73 | 0.05 |
| 8 | Dark enamel | -0.18 | 3.02 | 0.40 | 0.00026 | 0.00015 | 0.000022 | 0.000020 | 1.26 | 0.03 | 1.09 | 0.05 |
| 9 | Dark enamel | -0.06 | 2.97 | 0.24 | 0.00052 | 0.0002 | 0.000061 | 0.000025 | 1.22 | 0.05 | 0.96 | 0.07 |
| 10 | Dark enamel | 0.08 | 3.57 | 0.27 | 0.00045 | 0.00014 | 0.000041 | 0.000012 | 1.26 | 0.02 | 0.95 | 0.04 |
| 11 | Light enamel | 0.14 | 13.56 | 0.95 | 0.0107 | 0.0014 | 0.000257 | 0.000029 | 1.39 | 0.02 | 1.53 | 0.05 |
| 12 | Light enamel | 0.25 | 11.33 | 0.25 | 0.0169 | 0.001 | 0.000486 | 0.000025 | 1.42 | 0.01 | 1.69 | 0.02 |
| 13 | Light enamel | 0.41 | 11.86 | 0.15 | 0.0143 | 0.0017 | 0.000396 | 0.000049 | 1.46 | 0.01 | 1.72 | 0.02 |
| 14 | Light enamel | 0.53 | 9.21 | 0.29 | 0.0194 | 0.0014 | 0.000682 | 0.000035 | 1.42 | 0.01 | 1.80 | 0.02 |
| 15 | Light enamel | 0.64 | 9.34 | 0.18 | 0.029 | 0.0012 | 0.001017 | 0.000038 | 1.41 | 0.01 | 1.84 | 0.03 |
| 16 | Light enamel | 0.76 | 8.08 | 0.09 | 0.0402 | 0.0011 | 0.001638 | 0.000045 | 1.43 | 0.01 | 1.99 | 0.03 |
| 17 | Light enamel | 0.88 | 6.94 | 0.25 | 0.0638 | 0.0025 | 0.00304 | 0.00018 | 1.43 | 0.01 | 2.16 | 0.03 |
| 18 | Light enamel | 0.99 | 8.43 | 0.52 | 2.9 | 1.4 | 0.108 | 0.049 | 1.38 | 0.03 | 2.02 | 0.09 |
|  | MK16 coral |  | 2.58 | 0.14 | 0.000090 | 0.000060 | 0.0000085 | 0.0000091 | 1.111 | 0.011 | 0.759 | 0.019 |

**Table S6.** Uranium-series isotope data from spot analyses of *Stegodon* molar and 126 ka-old coral standard (MK16). Legend: 2SE = 2 standard errors on the mean calculated using Iolite™.

| Spot |  | Dimensionless distance | U (ppm) | 2SE | Th (ppm) | 2SE | (^232^Th/^238^U) | 2SE | (^234^U/^238^U) | 2SE | (^230^Th/^238^U) | 2SE |
| --- | --- | --- | --- | --- | --- | --- | --- | --- | --- | --- | --- | --- |
| 1 | Dentine | -0.9987 | 68.5 | 7.4 | 0.0184 | 0.0018 | 0.0000886 | 0.0000044 | 1.2475 | 0.0039 | 1.791 | 0.02 |
| 2 | Dentine | -0.8821 | 47.0 | 7.0 | 0.0071 | 0.0013 | 0.0000485 | 0.0000031 | 1.303 | 0.011 | 1.903 | 0.025 |
| 3 | Dentine | -0.7656 | 49.3 | 8.4 | 0.00593 | 0.00093 | 0.0000391 | 0.0000022 | 1.296 | 0.008 | 1.852 | 0.016 |
| 4 | Dentine | -0.6491 | 46.8 | 3.3 | 0.00454 | 0.00076 | 0.0000295 | 0.000002 | 1.3599 | 0.0067 | 1.946 | 0.018 |
| 5 | Dentine | -0.5326 | 51.7 | 6.1 | 0.00616 | 0.00073 | 0.000039 | 0.0000024 | 1.31 | 0.012 | 1.815 | 0.018 |
| 6 | Dentine | -0.4190 | 2.7 | 0.1 | 0.0002 | 0.0004 | 0.000023 | 0.00005 | 1.046 | 0.021 | 0.752 | 0.043 |
| 7 | Dark enamel | -0.2995 | 2.8 | 0.0 | 0.00032 | 0.00036 | 0.000037 | 0.000043 | 0.983 | 0.022 | 0.567 | 0.054 |
| 8 | Dark enamel | -0.1830 | 0.6 | 0.0 | Below LOD | Below LOD | -0.00005 | 0.00016 | 1.065 | 0.054 | 0.644 | 0.079 |
| 9 | Dark enamel | -0.0665 | 4.5 | 0.3 | 0.00046 | 0.00033 | 0.000034 | 0.000025 | 1.201 | 0.022 | 0.751 | 0.031 |
| 10 | Dark enamel | 0.0383 | 7.3 | 0.5 | 0.0023 | 0.0019 | 0.000109 | 0.000091 | 1.22 | 0.017 | 0.734 | 0.025 |
| 11 | Light enamel | 0.1971 | 11.0 | 1.1 | 0.0173 | 0.0011 | 0.000517 | 0.000019 | 1.47 | 0.016 | 1.855 | 0.025 |
| 12 | Light enamel | 0.3035 | 8.8 | 0.8 | 0.0191 | 0.0021 | 0.000706 | 0.000018 | 1.453 | 0.019 | 1.909 | 0.045 |
| 13 | Light enamel | 0.3996 | 10.2 | 1.4 | 0.0242 | 0.0029 | 0.000781 | 0.000019 | 1.425 | 0.013 | 1.954 | 0.036 |
| 14 | Light enamel | 0.5161 | 9.9 | 1.0 | 0.0317 | 0.0031 | 0.001047 | 0.000017 | 1.422 | 0.025 | 2.048 | 0.047 |
| 15 | Light enamel | 0.6327 | 8.4 | 0.8 | 0.0402 | 0.0046 | 0.001563 | 0.000051 | 1.429 | 0.023 | 2.11 | 0.1 |
| 16 | Light enamel | 0.7298 | 7.09 | 0.93 | 0.0753 | 0.0061 | 0.003671 | 0.00003 | 1.435 | 0.016 | 2.274 | 0.056 |
| 17 | Light enamel | 0.8337 | 5.51 | 0.4 | 0.134 | 0.013 | 0.0079 | 0.00029 | 1.452 | 0.014 | 2.31 | 0.071 |
| 18 | Light enamel | 0.9224 | 9.7 | 1.2 | 0.63 | 0.15 | 0.0205 | 0.0022 | 1.342 | 0.012 | 1.877 | 0.035 |
|  | MK16 coral |  | 3.17 | 0.30 | 0.00028 | 0.00020 | 0.000022 | 0.000017 | 1.106 | 0.008 | 0.772 | 0.017 |

**References**

Aplin KP, Helgen KM. 2010 Quaternary murid rodents of Timor. Part I: new material of *Coryphomys buelheri* Schaub, 1937, and description of a second species of the genus. *Bull Am Mus Nat Hist* **341**, 1-80.

Auffenberg W. 1988 *Gray’s monitor lizard*. Gainesville, FL: University of Florida Press.

Aziz F, van den Bergh GD, Morwood MJ, Hobbs D, Kurniawan I, Collins JG, Jatmiko. 2009 Excavations at Tangi Talo, central Flores, Indonesia. In *Pleistocene geology, palaeontology and archaeology of the Soa Basin, central Flores, Indonesia* (ed. Aziz F, Morwood MJ, van den Bergh GD), pp. 41-58. Bandung, Indonesia: Geological Survey Centre Bandung, Special Publication 36.

Bernal JP, Eggins SM, McCulloch MT. 2005 Accurate in situ 238 U–234 U–232 Th–230 Th analysis of silicate glasses and iron oxides by laser-ablation MC-ICP-MS. *J Anal At Spectrom* **20**, 1240-1249.

Brongersma LD. 1958 On an extinct species of the genus *Varanus* (Reptilia, Sauria) from the island of Flores. *Zool Med* **36**, 113-125.

Fabre PH, Pagès M, Musser GG, Fitriana YS, Fjeldså J, Jennings A, Jønsson KA, Kennedy J, Michaux J, Semiadi G, Supriatna N, Helgen KM. 2013 A new genus of rodent from Wallacea (Rodentia: Muridae: Murinae: Rattini), and its implication for biogeography and Indo-Pacific Rattini systematics. *Zool J Linn Soc* **169**, 408-447.

Forth G. 2008 *Images of the wildman in southeast Asia: an anthropological perspective.* Abingdon, UK: Routledge.

Grün R, Eggins S, Kinsley L, Moseley H, Sambridge M. 2014 Laser ablation U-series analysis of fossil bones and teeth. *Palaeogeogr Palaeoclimatol Palaeoecol* **416**, 150-167.

Hooijer DA. 1957 A *Stegodon* from Flores. *Treubia* **24**, 119-29.

Hooijer DA. 1981 What, if anything new, is *Stegodon sumbaensis* Sartono? *Mod Quaternary Res SE Asia* **6**, 89-90.

Hopkins SS. 2008 Reassessing the mass of exceptionally large rodents using toothrow length and area as proxies for body mass. *J Mammal* **89**, 232-243.

Kitchener DJ, Aplin KP, Boeadi. 1991a A new species of *Rattus* from Gunung Mutis, south west Timor Island, Indonesia. *Rec West Aust Mus* **15**, 445-461.

Kitchener DJ, How RA, Maharadatunkamsi. 1991b *Paulamys* sp. cf. *P. naso* (Musser, 1981) (Rodentia: Muridae) from Flores Island, Nusa Tenggara, Indonesia – description from a modern specimen and a consideration of its phylogenetic affinities. *Rec West Aust Mus* **15**, 171-189.

Kitchener DJ, How RA, Maharadatunkamsi. 1991c A new species of *Rattus* from the mountains of west Flores, Indonesia. *Rec West Aust Mus* **15**, 611-626.

Musser GG. 1981 The giant rat of Flores and its relatives east of Borneo and Bali. *Bull Am Mus Nat Hist* **169**, 67-176.

Musser GG, Newcomb C. 1983. Malaysian murids and the giant rat of Sumatra. *Bull Am Mus Nat Hist* **174**, 327-598.

Pagès M, Fabre PH, Chaval Y, Mortelliti A, Nicolas V, Wells K, Michaux JR, Lazzari V. 2016 Molecular phylogeny of south-east Asian arboreal murine rodents. *Zool Scripta* **45**, 349-364.

Sambridge M, Grün R, Eggins S. 2012 U-series dating of bone in an open system: the diffusion-adsorption-decay model. *Quat Geochronol* **9**, 42-53.

van den Bergh GD. 1999 The Late Neogene elephantoid-bearing faunas of Indonesia and their palaeozoogeographic implications*.* *Scripta Geolog* **117**, 1-419.

Zijlstra JS, van den Hoek Ostende L, Awe Due R. 2008 Verhoeven’s giant rat of Flores (*Papagomys theodorverhoeveni*, Muridae) extinct after all? *Contrib Zool* **77**, 25-31.
