# Supplementary material for: Quaternary vertebrate faunas from Sumba, Indonesia: implications for Wallacean biogeography and evolution
Source: Proc Biol Sci. 2017 Aug 30;284(1861):20171278. doi: 10.1098/rspb.2017.1278 (PMC5577490; doi:10.1098/rspb.2017.1278)
Supplement: Figure S1 [file rspb20171278supp2.pdf]

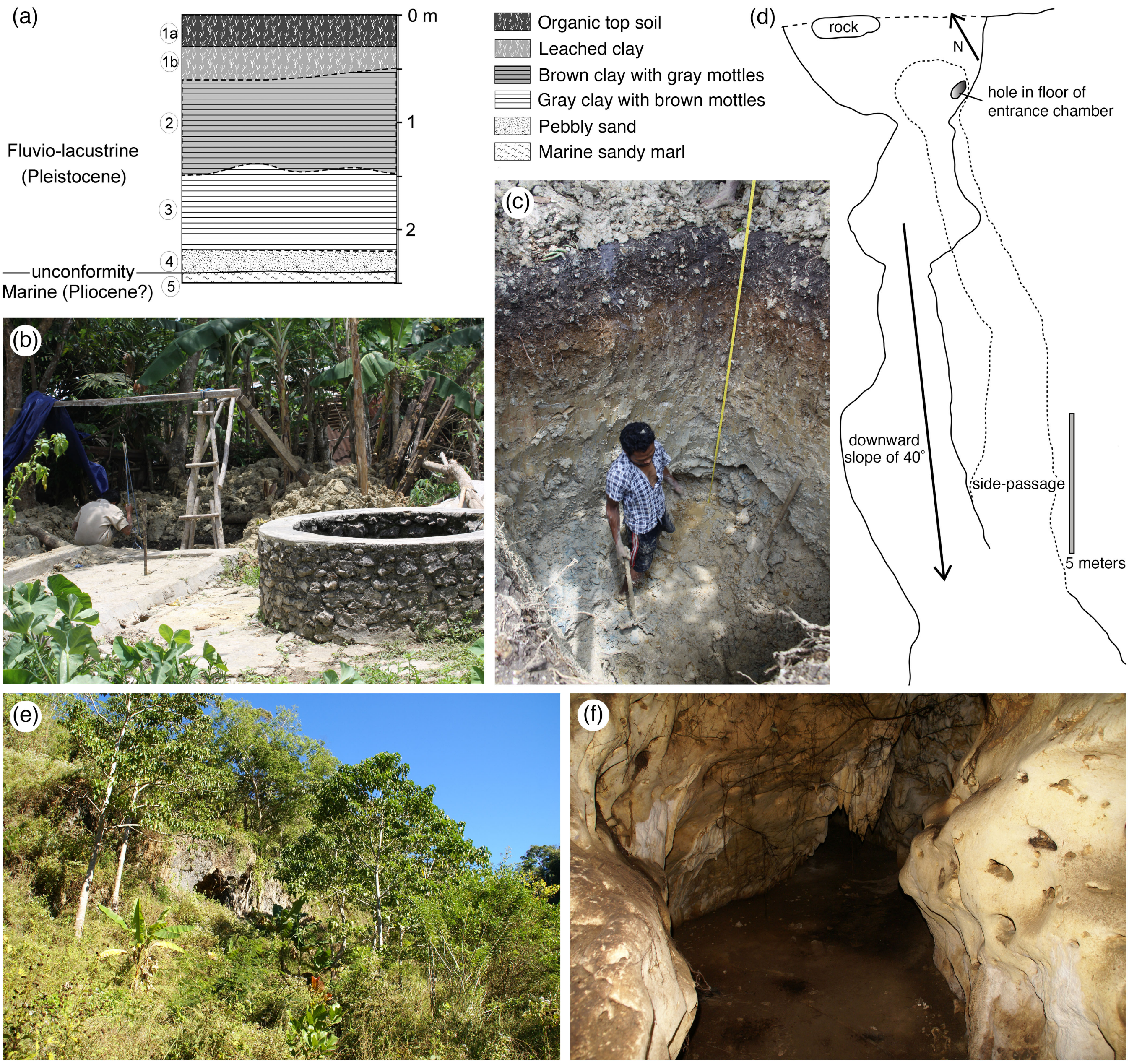

Fig. S1. Quaternary vertebrate fossil localities on Sumba. (a) Stratigraphic profile of the west baulk of the excavation at Lewapaku. Fossil vertebrate remains were concentrated in Layer 4. (b) View to the north at the Lewapaku excavation, with water well in foreground. (c) View towards northeast corner of Lewapaku excavation. (d) Plan view of Liang Lawuala. (e) Entrance to Liang Lawuala, looking southwest. (f) View to the northeast along the side-passage at Liang Lawuala, where fossil vertebrate remains were concentrated.
