# Supplementary material for: Quaternary vertebrate faunas from Sumba, Indonesia: implications for Wallacean biogeography and evolution
Source: Proc Biol Sci. 2017 Aug 30;284(1861):20171278. doi: 10.1098/rspb.2017.1278 (PMC5577490; doi:10.1098/rspb.2017.1278)
Supplement: Figure S2 [file rspb20171278supp3.pdf]

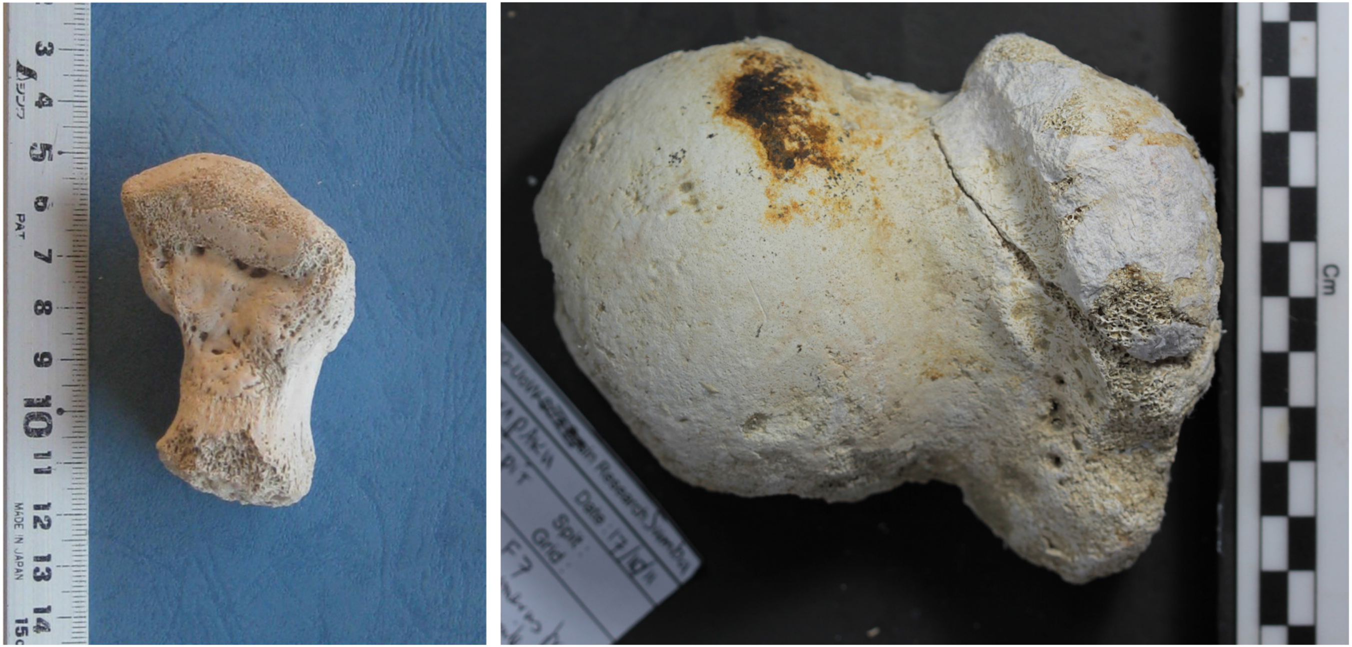

Fig. S2. Fossils of *Stegodon* from Lewapaku, Sumba. Left, metacarpus III, dextral, lateral view. Distal epiphysis is lacking. Right, proximal fragment of dextral humerus.
