# Supplementary material for: Quaternary vertebrate faunas from Sumba, Indonesia: implications for Wallacean biogeography and evolution
Source: Proc Biol Sci. 2017 Aug 30;284(1861):20171278. doi: 10.1098/rspb.2017.1278 (PMC5577490; doi:10.1098/rspb.2017.1278)
Supplement: Figure S3 [file rspb20171278supp4.pdf]

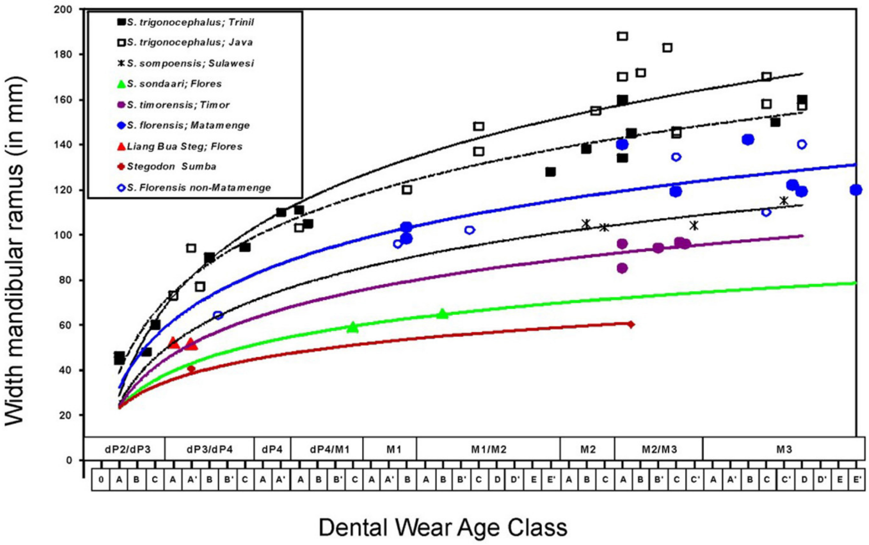

Fig. S3. Growth curves of *Stegodon* mandibles from various sites and of various species from Indonesia. Red triangles represent *S. florensis insularis* individuals from Liang Bua (only juvenile mandible known); red diamonds represent *Stegodon* mandibles from Sumba; green triangles represent *S. sondaari* from the Soa Basin (Flores).
