# Supplementary material for: Quaternary vertebrate faunas from Sumba, Indonesia: implications for Wallacean biogeography and evolution
Source: Proc Biol Sci. 2017 Aug 30;284(1861):20171278. doi: 10.1098/rspb.2017.1278 (PMC5577490; doi:10.1098/rspb.2017.1278)
Supplement: Figure S4 [file rspb20171278supp5.pdf]

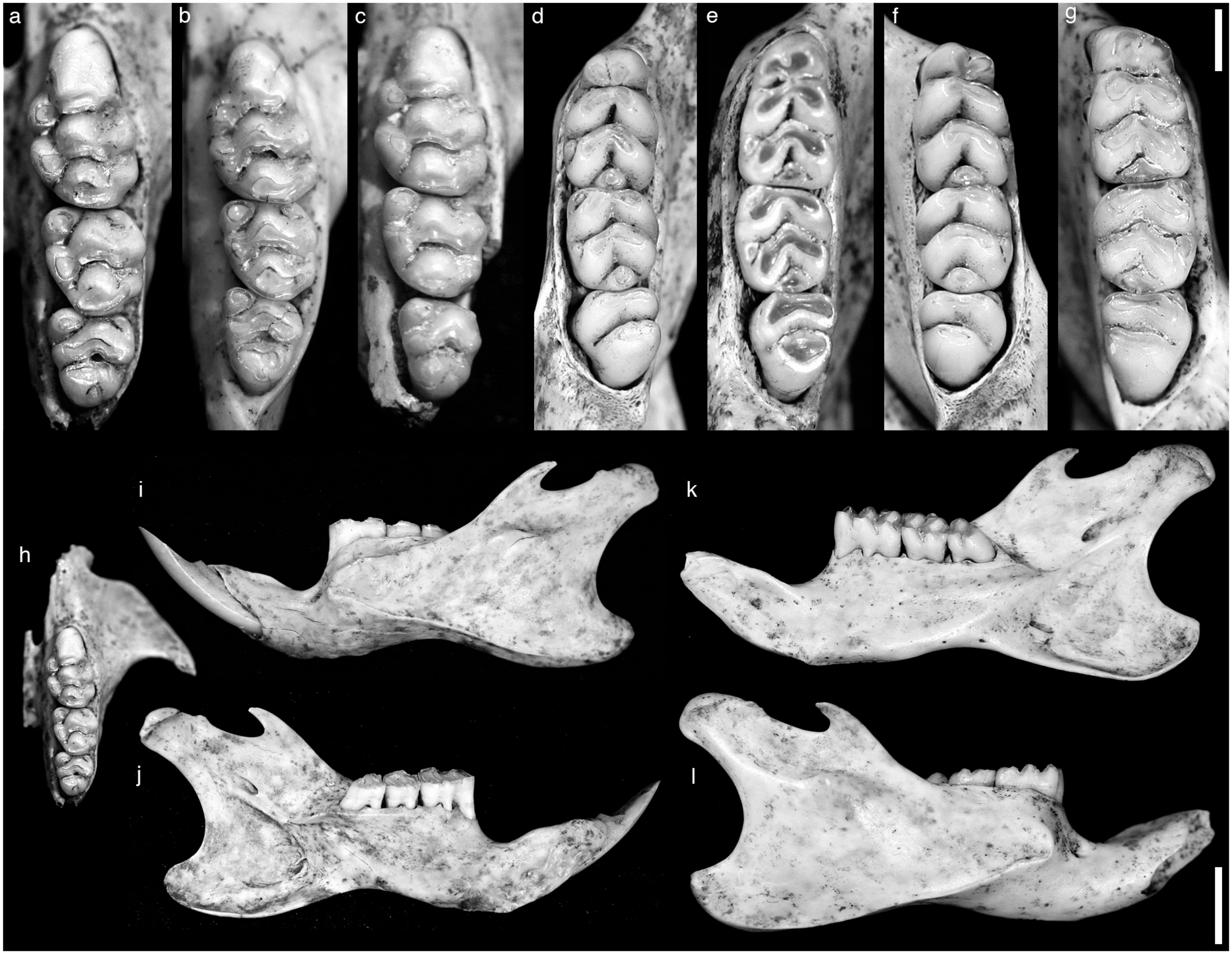

Figure S4. Maxillary and mandibular toothrows and cranial elements of *Milimonggamys juliae* gen. et sp. nov., from the late Holocene of Mahaniwa, Sumba. (a-c) maxillary toothrows: (a) LL 2014/2; (b) LL 2014/3; (c) LL 2014/4. (d-g) mandibular toothrows: (d) LL 2014/5; (e) LL 2014/6; (f) LL 2014/1 (holotype); (g) LL 2014/7. (h) LL 2014/2, maxilla. (i-l) hemimandibles: (i-j) LL 2014/8, left hemimandible, labial and lingual views; (k-l) LL 2014/7, right hemimandible, lingual and labial views. (a-g) scale bar = 2 mm; (h-l) scale bar = 5 mm.
