# Supplementary material for: Quaternary vertebrate faunas from Sumba, Indonesia: implications for Wallacean biogeography and evolution
Source: Proc Biol Sci. 2017 Aug 30;284(1861):20171278. doi: 10.1098/rspb.2017.1278 (PMC5577490; doi:10.1098/rspb.2017.1278)
Supplement: Figure S5 [file rspb20171278supp6.pdf]

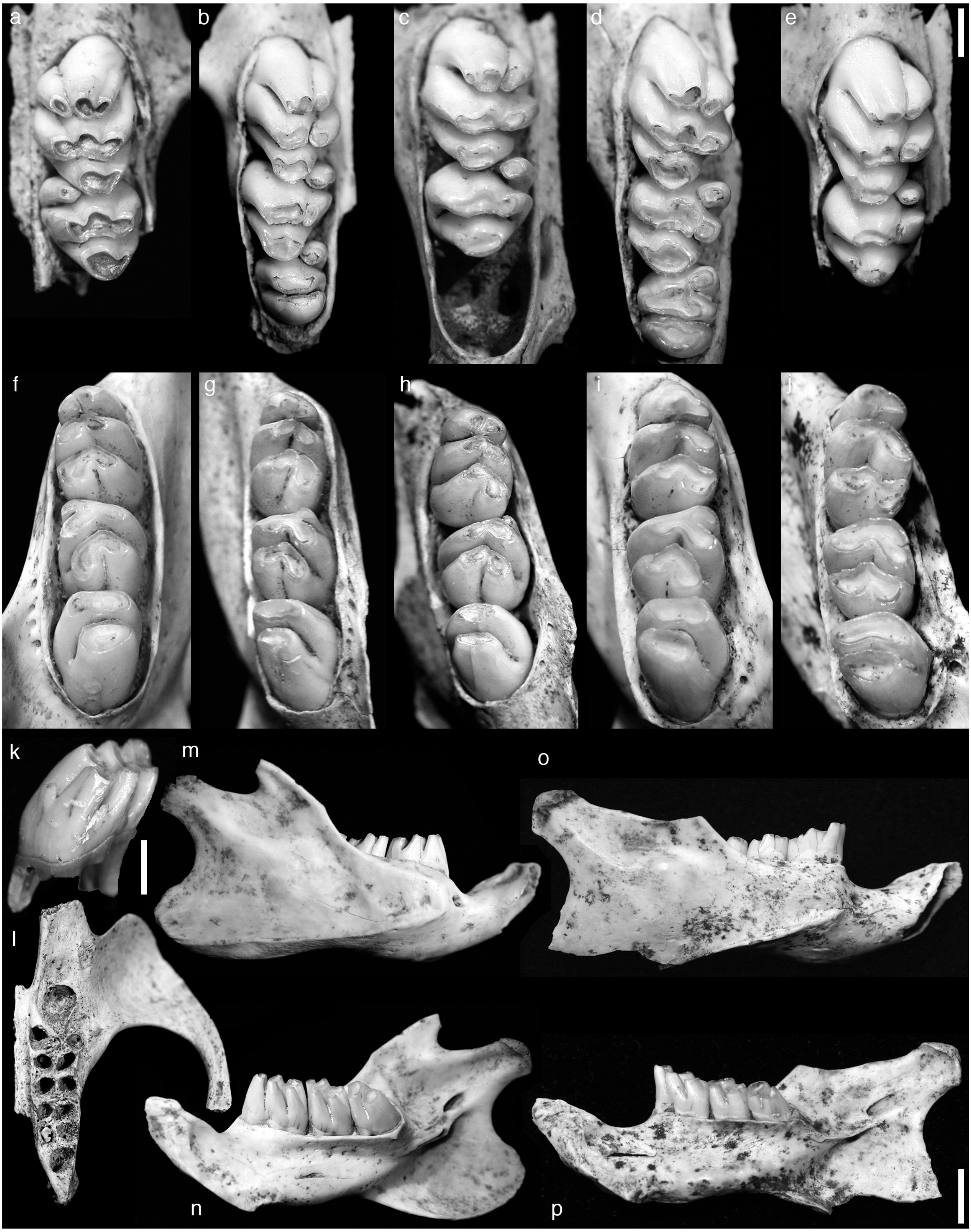


**Figure S5.** Maxillary and mandibular toothrows and cranial elements of *Raksasamys tikusbesar* gen. et sp. nov., from the late Holocene of Mahaniwa, Sumba. (a-e) maxillary toothrows: (a) LL 2014/10; (b) LL 2014/11; (c) LL 2014/12; (d) LL 2014/13; (e) LL 2014/14. (f-j) mandibular toothrows: (f) LL 2014/15; (g) LL 2014/16; (h) LL 2014/17; (i) LL 2014/9 (holotype); (j) LL 2014/18. (k) LL 2014/19, right m1 in posterolateral view, showing posterolabial cusplet and posterior cingulid. (l) LL 2014/20, edentulous maxilla. (m-p) hemimandibles: (m-n) LL 2014/9 (holotype), right hemimandible, labial and lingual views; (o-p) LL 2014/18, right hemimandible, labial and lingual views. (a-j) scale bar = 2 mm; (k) scale bar = 2 mm; (l-p) scale bar = 5 mm.
