# Supplementary material for: Quaternary vertebrate faunas from Sumba, Indonesia: implications for Wallacean biogeography and evolution
Source: Proc Biol Sci. 2017 Aug 30;284(1861):20171278. doi: 10.1098/rspb.2017.1278 (PMC5577490; doi:10.1098/rspb.2017.1278)
Supplement: Figure S6 [file rspb20171278supp7.pdf]

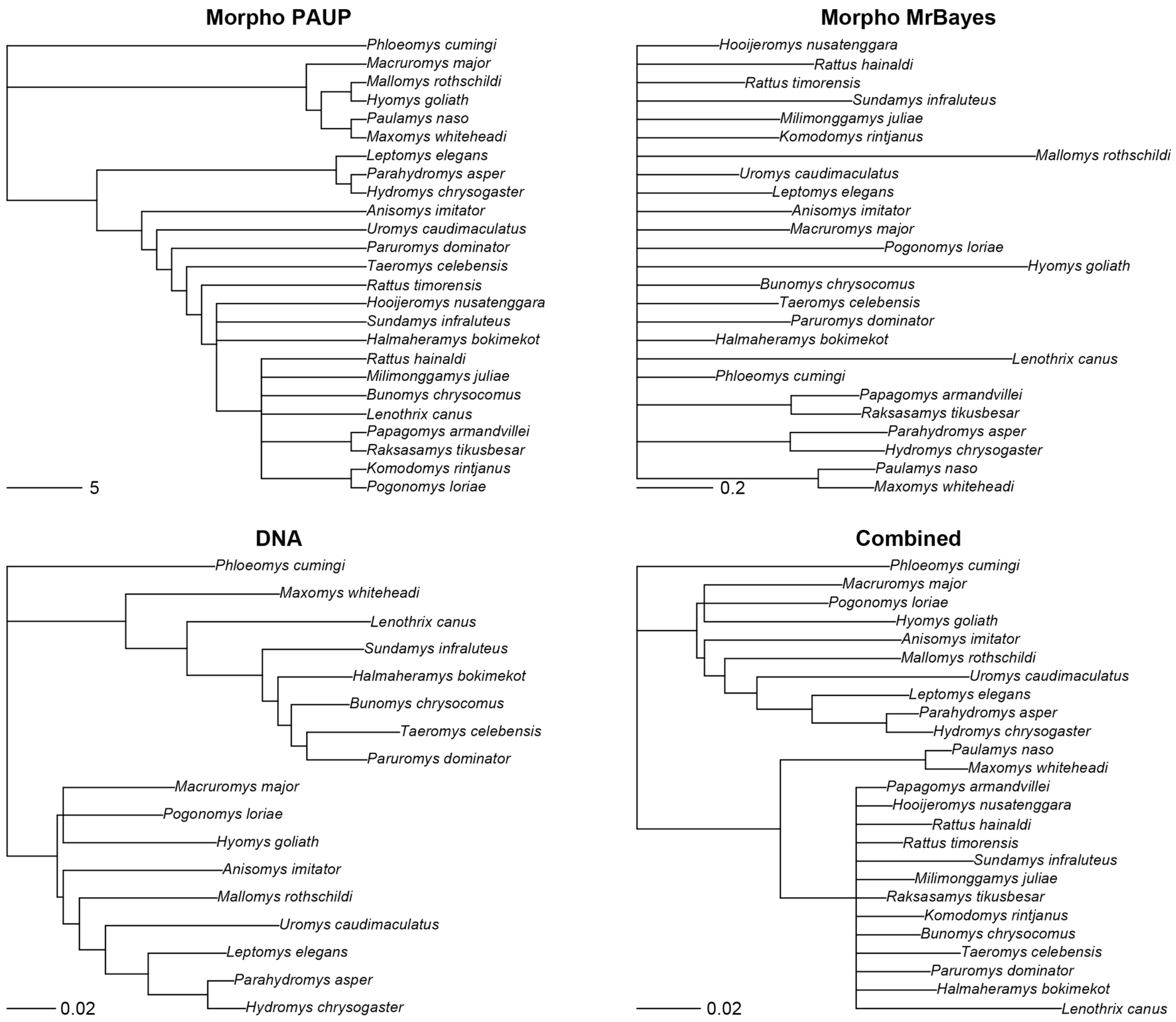

Fig. S6. Phylogenetic relationships of *Milimonggamys*, *Raksasamys*, and other southeast Asian and Australasian murids inferred from morphology-only data (using both parsimony and Bayesian analyses), molecular-only data, and combined morphological + molecular data. Scale bars indicate number of changes per unit distance.
