# Supplementary material for: Quaternary vertebrate faunas from Sumba, Indonesia: implications for Wallacean biogeography and evolution
Source: Proc Biol Sci. 2017 Aug 30;284(1861):20171278. doi: 10.1098/rspb.2017.1278 (PMC5577490; doi:10.1098/rspb.2017.1278)
Supplement: Figure S7 [file rspb20171278supp8.pdf]

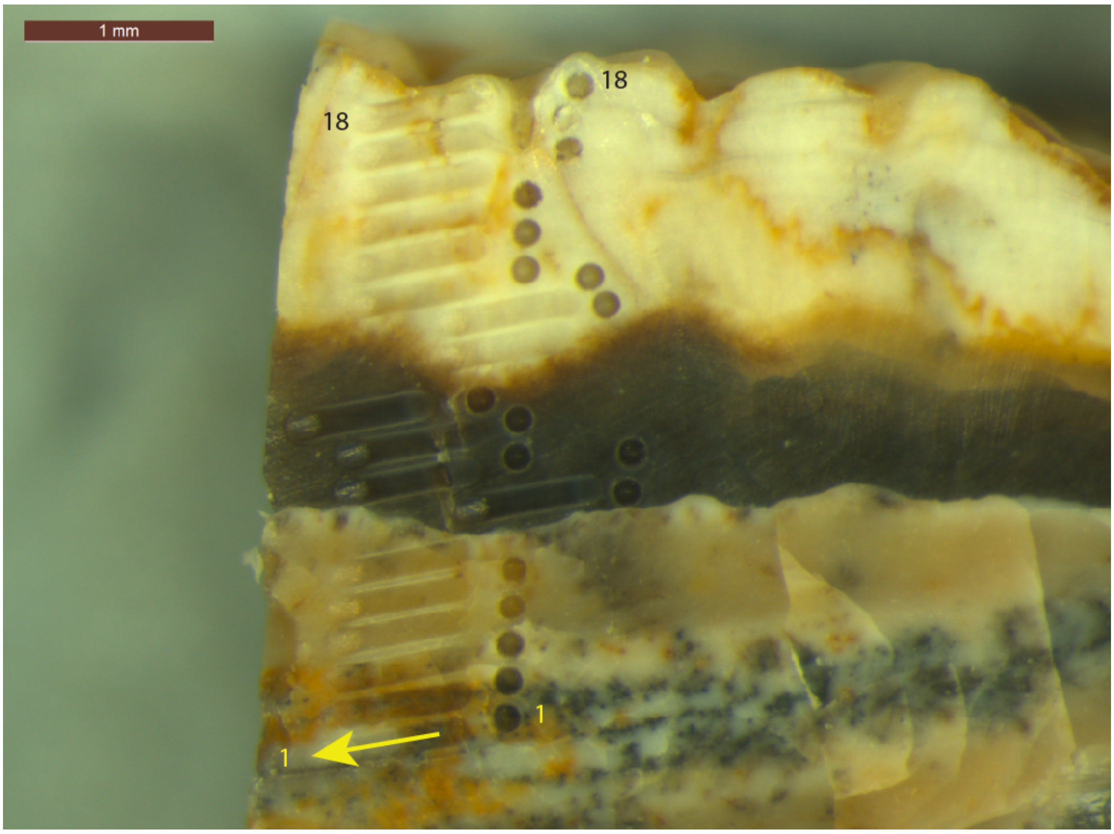

Fig. S7. Cross-section showing line and spot analyses (numbered 1 to 18; Table S4). Arrow shows direction of ablation for line analysis. For lines, the dimensionless distance is calculated using the coordinates of the start of the line. For line and spot analyses, the dimensionless distance of the boundary of each layer (including the surface) was calculated using the coordinates of the boundary just below or above lines or spots (i.e. boundaries have different coordinates whether considering line or spot analyses). A dimensionless distance of -1 refers to the centre of the dentine, which shows an axis of symmetry just below spot #1 and line #1.
