# Supplementary material for: Quaternary vertebrate faunas from Sumba, Indonesia: implications for Wallacean biogeography and evolution
Source: Proc Biol Sci. 2017 Aug 30;284(1861):20171278. doi: 10.1098/rspb.2017.1278 (PMC5577490; doi:10.1098/rspb.2017.1278)
Supplement: Figure S8 [file rspb20171278supp9.pdf]

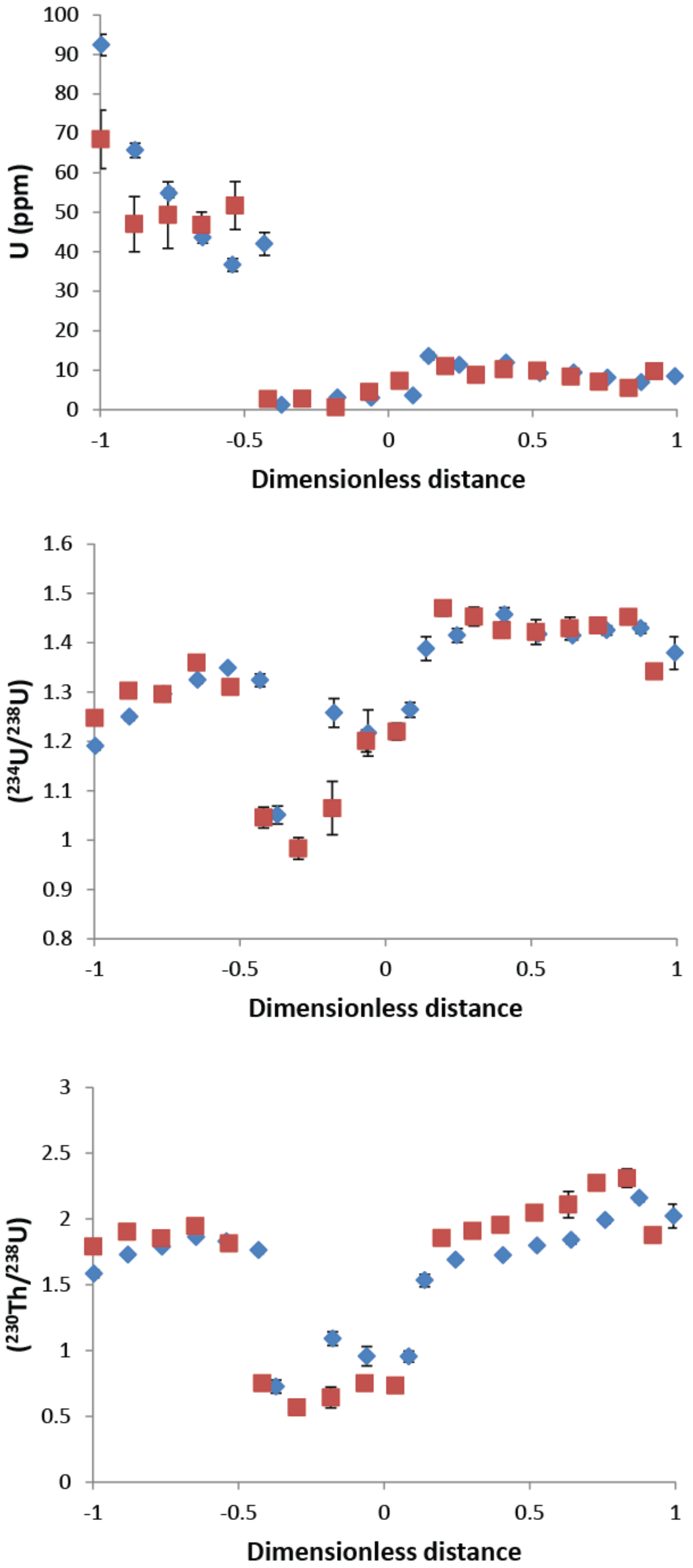

Fig. S8. U (in ppm), ($^{234}U/^{238}U$) and ($^{230}Th/^{238}U$) activity ratios in the *Stegodon* molar. Diamonds: line analyses; squares: spot analyses. A dimensionless distance of -1 corresponds to the centre of the dentine, while 1 represents the surface of the tooth.
